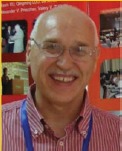# Recent progress in tissue optical clearing

**DOI:** 10.1002/lpor.201200056

**Published:** 2013-02-05

**Authors:** Dan Zhu, Kirill V Larin, Qingming Luo, Valery V Tuchin

**Affiliations:** 1 Britton Chance Center for Biomedical Photonics, Wuhan National Laboratory for Optoelectronics, Huazhong University of Science and TechnologyWuhan, China; 2 Key Laboratory of Biomedical Photonics of Ministry of Education, Department of Biomedical Engineering, Huazhong University of Science and TechnologyWuhan, China; 3 Department of Biomedical Engineering, University of Houston, Houston, USA and Department of Physiology and Biophysics, Baylor College of MedicineHouston, USA; 4 Department of Optics and Biophotonics, Saratov State UniversitySaratov, 410012, Russia; 5 Laboratory of Laser Diagnostics of Technical and Living Systems, Institute of Precise Mechanics and Control RASSaratov, 410028, Russia; 6 Optoelectronics and Measurement Techniques Laboratory, P.O. Box 4500, University of Oulu, FIN-90014Oulu, Finland

**Keywords:** tissue, scattering, optical-clearing agents, optical imaging, OCT, blood-flow imaging, microscopy imaging, molecular diffusion

## Abstract

Tissue optical clearing technique provides a prospective solution for the application of advanced optical methods in life sciences. This paper gives a review of recent developments in tissue optical clearing techniques. The physical, molecular and physiological mechanisms of tissue optical clearing are overviewed and discussed. Various methods for enhancing penetration of optical-clearing agents into tissue, such as physical methods, chemical-penetration enhancers and combination of physical and chemical methods are introduced. Combining the tissue optical clearing technique with advanced microscopy image or labeling technique, applications for 3D microstructure of whole tissues such as brain and central nervous system with unprecedented resolution are demonstrated. Moreover, the difference in diffusion and/or clearing ability of selected agents in healthy versus pathological tissues can provide a highly sensitive indicator of the tissue health/pathology condition. Finally, recent advances in optical clearing of soft or hard tissue for in vivo imaging and phototherapy are introduced.

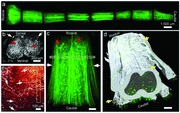

## 1. Introduction

Biomedical photonics is currently one of the fastest growing fields of life sciences connecting research in physics, optics and electrical engineering coupled with medical or biological applications. It allows structural and functional analysis of tissues and cells with resolution and contrast unattainable by any other method. However, the major challenges of many biophotonics techniques are associated with the need to enhance imaging resolution even further to the subcellular level as well as translate them for in vivo studies. Advanced optical methods combined with the various contrast agents pave the way towards real molecular imaging within living cells. However, the high scattering of turbid biological tissues limits the penetration of visible and near-infrared light, and the image blurs, both the imaging resolution and contrast decrease as light propagates deeper into the tissue 1, 2.

To perform deeper tissue imaging, several optical imaging techniques have been developed 2. For instance, the limit of imaging depth to 100 µm was broken with the invention of the multiphoton microscope 3. Combination of multiphoton fluorescence excitation with very high collection efficiency increased the depth of imaging in scattering samples up to 2 mm 4, 5. More recently, a multimodal molecular imaging technique has been introduced that combined light absorption with acoustic detection (thus termed photoacoustics) that further increased imaging depth up to a few centimeters 6. Alternatively, reducing scattering and absorption of tissues could also significantly enhance optical imaging of deep-tissue structures 7–11. For example, White used careful breeding techniques to create a transparent adult zebrafish, which has been applied to study cancer pathology and development in real-time 7, but this method is unsuitable for studies in human or other animals.

In contrast, a tissue optical clearing (TOC) method, proposed by Tuchin and coworkers, using immersion of tissues into optical-clearing agents (OCAs) that reduces the scattering of tissue and make tissue more transparent 8–12. It is well known that tissues are densely packed with many types of substances, including scattering particles with higher refractive index, i.e. collagen, elastic fibers, cells and cell compartments, and surrounding media with lower refractive index, i.e. interstitial fluid and/or cytoplasm. This architecture makes light travel at different speeds and angles because each component has a different refractive index. Typical OCAs usually have high refractive index and, thus, penetration of OCAs into the extracellular space matches the refractive indices of the scatters and their surrounding media, and then lead to reduction of the light scattering. This process leads to significantly enhancement of the imaging depth as light penetrates deeper into the tissues 11.

After the concept of TOC was introduced, several groups were actively investigating the possibility of tissue optical clearing and demonstrated the validity for improving optical imaging depth or contrast 13–31. Many new original studies are elaborated using TOC in combination with laser speckle contrast imaging (LSCI) 32–34, optical coherence tomography (OCT) 35–42, microscopy imaging 43–51, ultramicroscopy 51–53, etc., which have demonstrated a great power not only for tissue structural and functional imaging with higher resolution 46–54, but also for optical imaging and diagnosis in vivo 33, 34, 55. In addition, when the OCT technique is applied to realize the time- and depth-resolved monitoring of OCAs diffusion, it might be possible to distinguish between normal and pathological tissues 56–69.

In order to find more optimal OCAs, the mechanisms of TOC have been carefully investigated from the macroscopic (e.g. tissues, tissue phantoms) to microscopic (molecular) level 70–78 and at both in vitro and in vivo studies 79–83. Various physical methods, such as light irradiation 84–86, light fractional ablation 87, 88, ultrasound application 89–92, sandpaper grinding 92, microneedle rolling 93, 94 and mechanical compression 95–99, or chemical-penetration enhancers 99–110 were used to enhance the delivery of OCAs into tissue, so as to improve the tissue optical-clearing efficacy. This paper focuses on an overview of recent progress in the development and applications of TOC technique, which includes mechanisms of TOC, methods for enhancing TOC efficacy and applications of TOC for tissue optical slicing. Finally, in vivo applications and the safety of the TOC technique will be analyzed and discussed.

## 2. Mechanisms of TOC

The diffusion of optical-clearing agents with higher refractive indices and higher osmolality into tissues will match the refractive indices of tissue components with extracellular fluid, thus reducing the scattering of tissue. This was regarded as the major mechanism of TOC 8–12. However, in vitro experiments demonstrated that optical-clearing efficacy of skin did not correlate with refractive indices of OCAs, but with the molecular structure of OCAs 71, 72, which was further supported by molecular dynamics simulation 78. Furthermore, additional mechanisms such as dissociation of collagen fibers 73–77 and tissue dehydration 78–82 caused by OCAs have been proposed to explain the mechanism of TOC based on experimental results. However, for in vivo tissue optical clearing, only the thickness of dermis and the diameter of collagen fiber were reduced, but there was no dissociation of collagen fiber as observed during in vitro skin optical clearing studies 83. Different views on the mechanisms of optical clearing are likely due to different experimental protocols and techniques as well as difference in studied clearing agents and the complex of mechanical and chemical properties for different tissues.

### 2.1. Physical mechanism of optical clearing

In order to eliminate the effect of tissue inhomogeneity, Wen et al. performed studies on a simple tissue-simulating phantom based on Intralipid 70. Intralipid is a homogeneous fat emulsion consisting of lipid droplets dissolved in water. Even though it has no collagen structure like tissue, it is a commonly used compound that simulates tissue optical properties 1. They mixed six different common OCAs, such as DMSO, glycerol, 1,4-butanediol, 1,2-propanediol, PEG200, PEG400 and water with 10%-Intralipid, respectively, while keeping the concentration of scattering particles of the mixtures constant. The results demonstrated that 5% Intralipid without any OCAs completely conceals a pattern under the sample box, while the addition of OCAs can make the Intralipid transparent at different degrees, and the pattern became visible. Also, after adding PEG200 and PEG400, the mixture becomes more transparent but nonuniform due to aggregation of particles. The other four OCAs can keep the mixture uniform, and the optical-clearing efficacy decreases gradually for DMSO, glycerol, 1,4-butanediol, 1,2-propanediol, respectively, whose refractive indices reduce gradually.

**Figure 1:**
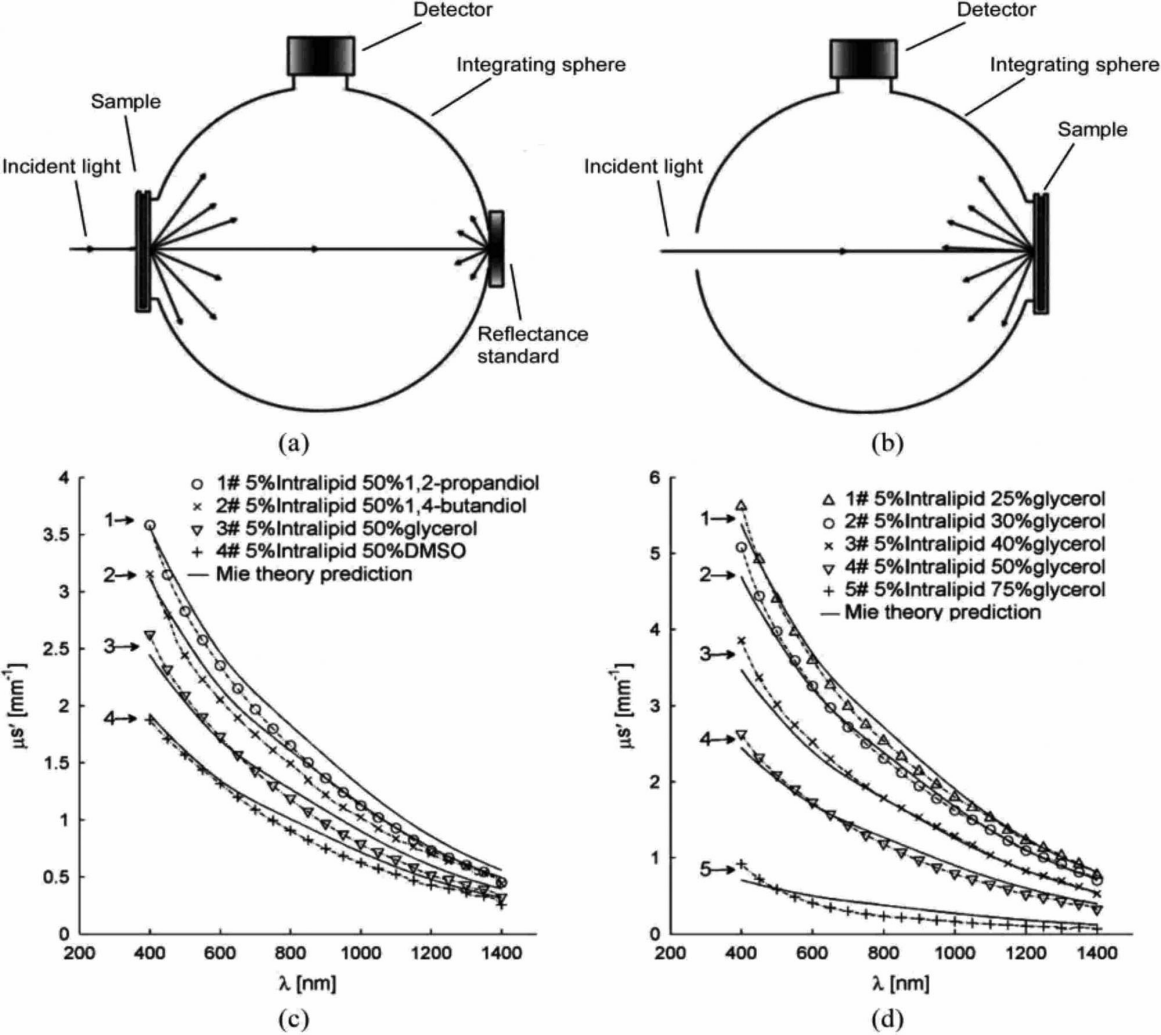
Schematic diagram of integrating sphere for measuring (a) transmittance and (b) reflectance; the comparison between the theory predicted reduced scattering coefficients and the experimental measured results: (c) 5%-Intralipid with 50%-OCAs (DMSO, glycerol, 1,4-butanediol, and 1,2-propanediol); (d) 5%-Intralipid with 75%-, 50%-, 40%-, 30%-, 25%-glycerol 70.

Additionally, the reduced scattering coefficients of uniform mixtures were estimated with Mie theory based on the particle-size distribution measured by an electron microscopy and the refractive index of the background medium. Also, a commercially available spectrophotometer (Lambda 950, PerkinElmer, USA) with an integrating sphere of 150 mm diameter was used to measure the transmittance and the reflectance of the sample and then the inversed adding-doubling (IAD) algorithm 111 was used to calculate the reduced scattering coefficient. Figures 1a and b show the schematic diagram of the integrating sphere for measuring the reference or transmittance 112. Figures 1c and d demonstrate that the theoretical calculations match well to the experimental results for different OCAs, which has a good consistency with direct observation 70.

Therefore, the mechanisms of TOC could be due to two aspects: one is the interaction between OCAs and tissue phantom, which makes the phantom more transparent but nonuniform from the direct observation; and the other is the enhancement of refractive index of the background medium caused by OCAs according to the theoretical estimation and measurement for the uniform mixture.

### 2.2. Molecular mechanism of optical clearing

Various biocompatible chemical agents were applied to study the optical-clearing effect of tissues 8–12. These agents can be broadly classified as alcohols (glycerol, polyethylene glycol (PEG), butanediol, TMP, the combined PPG-and PEG-based prepolymer mixture, sorbitol, xylitol) 71, 72, sugars (glucose, dextrose, fructose, sucrose) 17, 18, organic acid (oleic acid) 17, 71, and other organic solvents (DMSO) 13, 17, 71.

Choi et al. used optical clearing potential (OCP), the ratio of reduced scattering coefficient of tissue measured before and after OCAs application, to quantitatively evaluate the tissue optical-clearing efficacy caused by chemical agents 71. In order to understand the dependence of OCP on OCAs, they measured the refractive indices and osmolality (concentration in units of osmole per kilogram of water) of 12 agents. After having OCAs contacted with the epidermal side of human skin in vitro, they found that the OCP does not correlate with the refractive index or with the osmolality of OCAs. It was also found that both hydrophilic hydroxyl-terminated prepolymer and hydroxyl-terminated chemical agents have better OCP.

Further, Mao et al. 72 focused on a study of six alcohols with hydroxy-terminated organic compounds (1-butanol, 1,4-butanediol, 1, 3-propanediol, PEG200, PEG400, and glycerol) whose molecular weight ranges from 74 to 420 Da and the refractive index ranges from 1.40 to 1.47. In order to better simulate the topical application of OCAs to the skin in vivo, OCAs were topical applied to the epidermis and saline to the dermis of porcine skin in vitro, and the relative transmittance measured with an integrating sphere was used to evaluate the optical-clearing effect 85. They found that glycerol, triatomic alcohol, caused the greatest optical-clearing effect, 1-butanol, monohydric alcohol caused the poorest effect, and the four diatomic alcohols produced moderate response. After examining the relationship between the optical-clearing efficacy and the refractive index or the molecular weight of these OCAs, the authors concluded that the optical-clearing effect of skin induced by alcohols should be related to the number of hydroxyl groups, but not to the refractive index or the molecular weight of optical-clearing agents.

Collagen fibers have complex self-assemble structures and are the major scattering centers in tissues 75. They are widely distributed in different tissues and especially abundant in the dermis of the skin and sclera of the eye. In spite of nearly 30 years of research, there is still no of exact explanation for how collagen complex structure forms. However, it is well established that hydrogen bonding is the primary bonding force between collagen triple helices. OCAs with multiple hydroxyl groups have strong electronegativity, which destabilizes the higher-order structure of collagen to its dissociation. As the screen of hydrogen bonds in collagen triple helices is powered by a noncovalent interaction, the OCAs effect on the dissociation of collagen can be relatively easy reversible. Yeh et al. 73–76 observed the dissociation of collagen fibers for in vitro immersion of tissues in glycerol, while removal of excess glycerol and subsequent application of PBS led to a recovery of the collagen fibrous structure.

Hirshburg et al. 77 performed molecular dynamic (MD) simulation to explore the formation of hydrogen bonds between alcohols (glycerol, xylitol and sorbitol) and collagen molecules. They divided the hydrogen-bond bridges into different types by the positions of the participating hydroxyl groups on carbon atoms. The index of types is related to the number of carbon atoms of alcohols in a hydrogen-bond bridge. They found that the higher bridge types span further across the collagen surface, and can potentially disrupt collagen–collagen and collagen–water interactions more efficiently than lower bridge types. Thus, alcohols with hydroxyl group pairs with longer distance on the carbon chain should have better optical-clearing efficacy than alcohols that have only hydroxyl groups next to each other. The simulation results provided a good explanation for the fact that 1,3-propanediol was shown to have twice the OCP of 1,2-propanediol even though they had identical molecular weights (76.10 Da), similar refractive index (1.44 versus 1.43), and osmolality (8.3 versus 8.7 Osm/kg) (1,2-propanediol can form only type I bridges, while 1,3-propanediol forms only type II bridges) 77. Since the MD simulation can reveal the details of interaction between OCAs and tissue structure on the microscopic (molecular) scale, it can be a powerful tool in exploring the mechanisms of TOC and the screening for new highly efficient optical-clearing agents.

### 2.3. Dehydration during TOC

Besides the ability of OCAs to match the refractive indices of tissue components, tissue dehydration can be an important mechanism of TOC 78–82. Application of a hyperosmotic OCA to the tissue surface induces water flux from the interstitial space to the tissue surface and even out of the tissue, increases the osmolality of interstitial fluid (ISF), and, consequently, draws water from cells and/or collagen fibers. These processes give an additional refractive-index matching effect due to reduction of water content in the interstitial space, and reduce the overall thickness of tissue and make it denser (more ordered). All these effects raise the optical transmittance and reduce diffuse reflectance of the tissue. Indeed, the dehydration of tissue commonly happens during the optical-clearing process.

**Figure 2:**
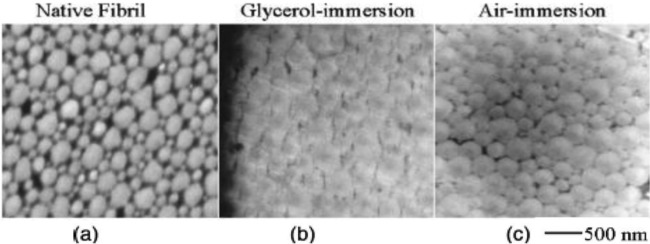
TEM images of tail tendon fibrils: native state (a); 20-min glycerol-immersion (b); 2-h air-immersion (c) 78.

Actually, other physical methods, i.e. extrusion or flattening also induce tissue dehydration without application of OCAs. After having measured the changes in optical transmittance of rat skin through application of DMSO and glycerol or by an air-dried method, Rylander et al. found that the dehydrated samples get an enhancement of light transmittance similar to that induced by OCAs treatment, but it takes longer time to achieve equal effect 78. Figure 2 shows the ultrastructure details of tail tendon fibrils from a transmission electron microscopy (TEM). Images reveal that the changes induced by OCAs immersion are similar to that by dehydration, and there is higher fibril packing density in the glycerol and air-immersed state versus the native state. The volume fraction of tendon fibrils increased from 0.65 to 0.90 due to glycerol immersion and air dehydration. The measured increase in scattering particle volume fraction corresponds to an approximately 60% decrease in reduced scattering coefficient assuming no change in refractive-index ratio between fibrils and surrounding fluid or change in scattering particle size or shape. Therefore, this study suggests that ISF concentration in tissue play important role in the clearing process.

The human skin dehydration and hyperosmotic optical-clearing agent delivery through epidermis at both intact and perforated (lamp fractional ablation) stratum corneum (SC) is described in Ref. 80. Accelerated diffusion of water and 88%-glycerol in aqueous solution was found as a result of enhanced epidermal permeability. The dehydration process induced by both stimulated evaporation (air drying) and osmotic agent action was studied by weight measurements with concurrent monitoring of refractive-index alteration of both glycerol solution and water during their interaction with skin samples. The amount of water escaping from skin through the SC due to glycerol action as a hyperosmotic agent and the amount of glycerol penetrating through the skin sample were also estimated 80. This is possible due to the osmotic driving force of glycerol that due to its hygroscopic properties produces a water flux from in depth of skin via living epidermis and SC outside the skin 113. It was shown that skin surface fractional microablation reduces the SC barrier function and promotes water escape from the skin and penetration of a hyperosmotic agent into skin 80. The study of evaporative process allows separation of the dehydration mechanism of optical clearing from other mechanisms, i.e. refractive-index matching or structural modification of the collagen. From the practical point of view, topical admission of OCAs into natural skin prevents the formation of high concentration gradients between interstitial liquid and applied solution that decreases the dehydration of tissue and can be considered as a positive factor for in vivo skin optical clearing, since dehydration relates to decreasing the rate of the fluid-exchange process.

Optical spectroscopy can be employed to quantitatively analyze the water loss during the optical-clearing process. Having considered the strong absorption bands of water in the near-infrared region, Wang and coworkers used dual-wavelength analysis method to estimate the water content of skin based on diffuse reflectance spectra at *λ*_1_ = 1100 and *λ*_2_ = 1936 nm 17–19. The authors demonstrated that the changes in water content of skin show good consistency with optical-clearing effect, which quickly decreased in the first minute and then became slower under immersion in 80%-glycerol or 80%-propanediol.

**Figure 3:**
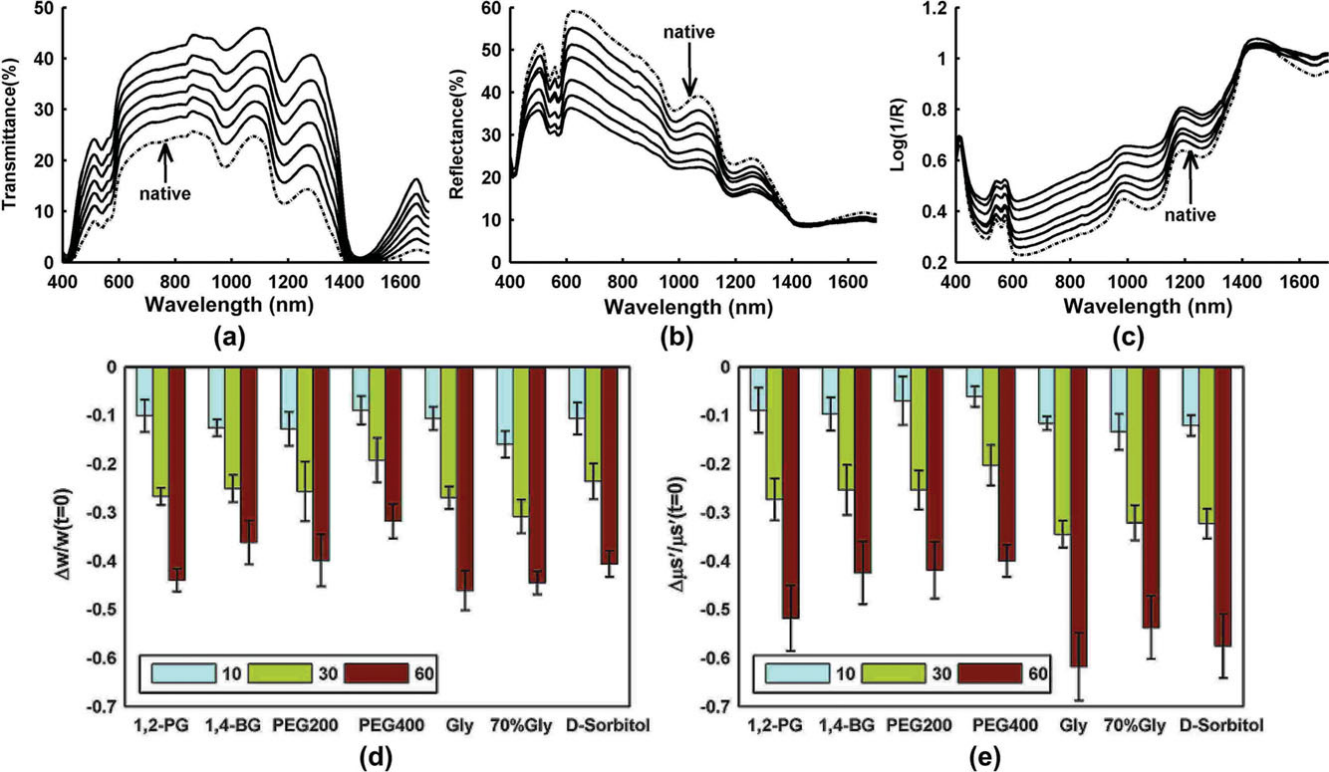
(online color at: http://www.lpr-journal.org) Spectral changes for the skin sample before and after treatment with 1,2-propanediol, over the wavelength range of 400–1700 nm: (a) Transmittance after treatment by the agent at time intervals of 0, 10, 20, 30, 40, 50, and 60 min (from bottom to top); (b) corresponding reflectance spectra (from top to bottom); (c) corresponding apparent absorbance (log 1/R) (from bottom to top); and relative reduction of water content (d) and reduced scattering coefficient (e) after 10-, 30-, and 60-min treatment with the OCAs 81.

Yu et al. 81 developed a PLS regression model based on reflectance spectra in the range from 1100 to 1700 nm to quantitatively investigate the dehydration of skin. By using a commercially available spectrophotometer (Lambda 950, PerkinElmer, USA) with an integrating sphere, the dynamical reflectance and transmittance spectra of samples before and after treatment of OCAs at time intervals of 0, 10, 20, 30, 40, 50, and 60 min were measured, respectively. It can be seen in Figs. 3a and b that the typical transmittance increases with the time over the wavelength range (400–1700 nm) after treatment by 1,2-propanediol, while the reflectance decreases in the range from 400 to 1370 nm and increases from 1370 to 1700 nm. The corresponding apparent absorption (log 1/*R*) is shown in Fig. 3c. The reduced scattering coefficients at different time intervals were also calculated using the IAD algorithm based on the measurements of reflectance and transmittance, and the water content was calculated by the apparent absorption. Figures 3d and e show the relative changes in scattering coefficients and water content at 10, 30, and 60 min after immersion of six different alcohol OCAs. It can be seen that both the water content and reduced scattering coefficient decrease upon the treatment with OCAs. Statistical analysis indicates that upon topical application of glycerol, or D-sorbitol, there was an extremely significant difference between the relative changes in reduced scattering coefficient and water content (*P* < 0.01), and a significant difference for PEG 400 (*P* < 0.05). On the contrary, for 1,2-propanediol, 1,4-butanediol, or PEG200, there was no statistical difference between the two sets of data (*P* > 0.05). The authors concluded that dehydration is the main mechanism of skin optical clearing for 1,2-propanediol, 1,4-butanediol, or PEG200 during the 60-min topical treatment, whereas for PEG400, glycerol, or D-sorbitol, there should exist other mechanisms that lead to further clearing besides the dehydration.

### 2.4. In vivo mechanism of TOC

Since there are significant differences between structure and function of tissues under the in vivo and in vitro conditions, it needs to be further discussed whether there are differences between in vivo and in vitro mechanisms of TOC.

Wen et al. 83 did preliminary work on exploring in vivo mechanisms of skin optical clearing by glycerol solution. Optical clearing efficacy was achieved by dermal injection of 20%-, 30%-, or 75%-glycerol solution on the dorsal skin of Sprague−Dawley (SD) rats. They found that after injection of glycerol, the reflectance spectrum of skin decreases quickly in the range of 400−900 nm. The higher the concentration of glycerol used, the larger the observed reflectance decreases. With increased time of the experiments, the reflectance increases again. The skin structure was examined by hematoxylin-and-eosin (H&E) histological analysis, electron microscopy (EM) imaging and second-harmonic generation (SHG) imaging at 10 min after injection of glycerol, as the optimal time.

**Figure 4:**
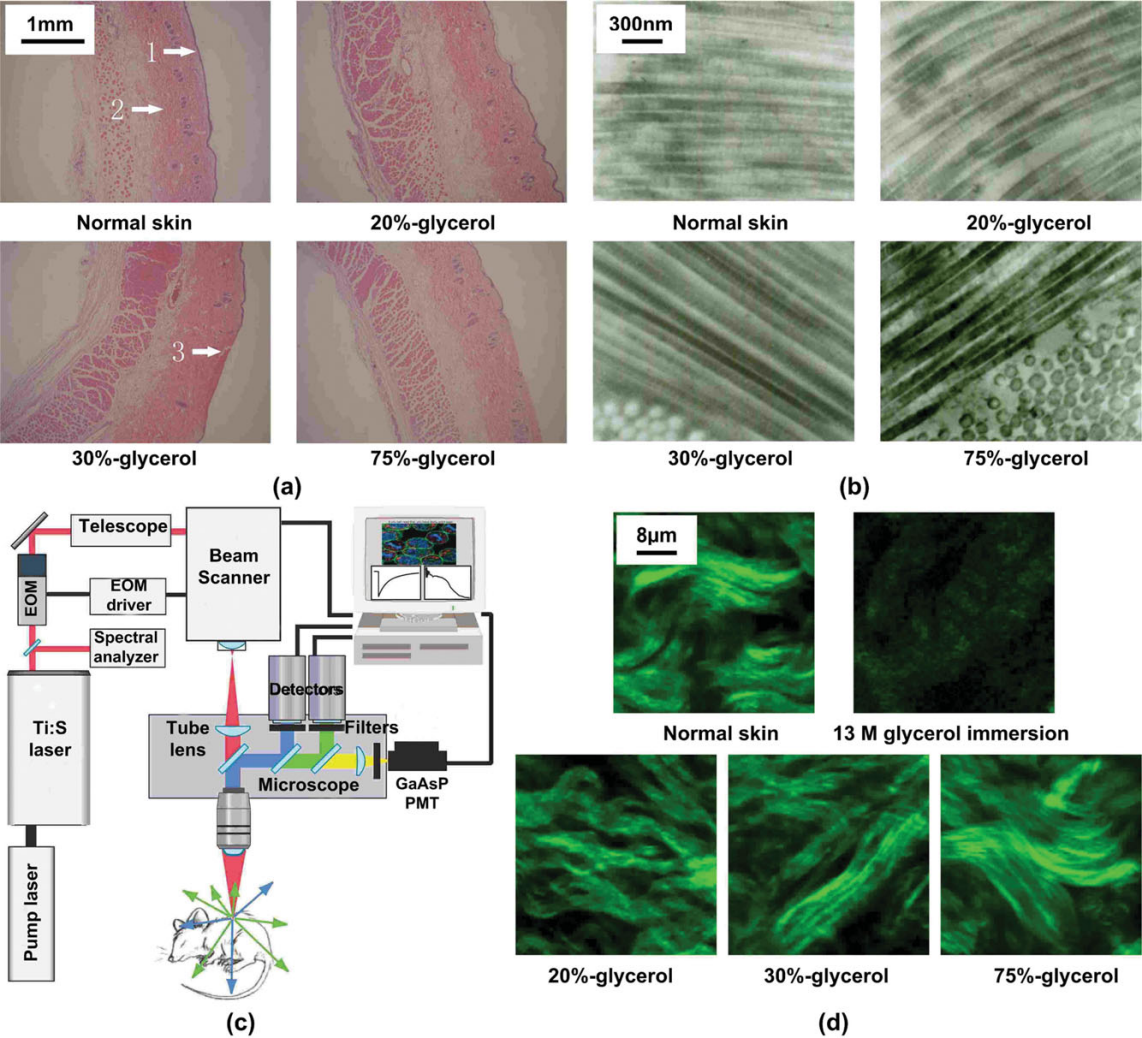
(online color at: http://www.lpr-journal.org) Intact skin sample and 10 min after injection of 20%-glycerol, 30%-glycerol, or 75%-glycerol; (a) HE-histology; (b) EM images; (d) SHG images 83; (c) schematic diagram of typical TPEFM for SHG imaging.

Figures 4a and b show the microstructure and ultra-microstructure of skin, respectively. The results indicate that the thickness of dermis becomes less with the increase of glycerol concentration. The collagen fibers in all groups of measurements are arranged in order. However, 75%-glycerol injection significantly reduces the diameter of collagen fibers, whereas for other groups there are hardly seen any changes in collagen diameters compared with the intact sample.

The collagen-fiber structure of skin is an important source of the second-harmonic generation (SHG) signal. In order to explore the effects of OCAs on collagen-fibers, a two-photon excitation fluorescence microscopy (TPEFM) was applied to image in vitro skin after treatment (see Fig. 4c. A Ti: sapphire laser generating 79- nm, 100-fs, 80-MHz pulses is coupled into an inverted microscope (FV1000, Olympus, Japan). To isolate the SHG signal, a bandpass filter at 395 nm (395/11, Sigma, USA) was placed in front of the photomultiplier tube. The image integration time was 12.5 ms/pixel. Figure 4d show the SHG images of collagen fibers in dermis. It can be observed that 10 min after injection of glycerol of different concentrations, the SHG signal is still strong, which means there is no collagen dissociation. However, the results for ex vivo are different from those for in vitro skin that was immersed in 13 M glycerol for 30 min, in which the SHG signal becomes much weaker and the bundled pattern can no longer be observed in SHG images due to collagen dissociation 73, 74.

It has been suggested that the interaction between collagen and OCAs screens noncovalent attractive forces resulting in inhibition of collagen self-assembly in solution and destabilization of high-order structures in skin 73, 74. The change in collagen structure and size can lead to a substantial reduction in tissue light scattering. Therefore, it was concluded that the collagen dissociation is an important mechanism of in vitro TOC 73, 74, but the decrease in size is that of in vivo TOC 73. Different results could be due to a difference between in vivo and in vitro skin, i.e. the metabolism takes away some agent and decreases the local concentration of glycerol.

Summarizing, it is clear that the mechanisms of TOC could be due to multiple factors: OCAs increase the refractive index of interstitial fluid and lead to a refractive indices match among of various substances in tissue; hyperosmotic agents induce dehydration of tissue, decrease the thickness of tissue; molecular dynamical reactions between tissue and OCAs cause dehydration of tissue or change tissue structure temporarily. Since the structure and components of tissues or OCA_S_ different, the TOC process could also be different.

## 3. Enhancement of TOC

As discussed above, the TOC efficacy depends not only on the type of OCAs but also on its treatment time. The longer tissue is immersed, the more transparent the tissue becomes 72. For instance, it usually takes several weeks, even several months to make a mouse brain transparent when immersed into OCA such as Scale 51. Compared with other soft tissues, it is more difficult to induce clearing of the skin by topical treatment with OCAs because the barrier function of stratum corneum restricts the penetration of OCAs into the dermis. For in vivo applications of the optical clearing technique, the direct exposure of skin dermis to OCAs or dermal injection of OCAs are required. However, OCAs at low concentration cannot make skin sufficiently optically cleared, while OCAs at high concentration could induce edema, suppuration, or even scarring 15, 114. In order to develop an effective and safe way to breach the SC integrity and accelerate the permeability of OCAs into dermis, various physical methods 84–99, chemical enhancers 100–108, and their combinations 109, 110 have been introduced.

### 3.1. Physical enhancement

A number of mechanically related options were utilized to breach the SC barrier function, such as intensive pulsed light (IPL) 84–86, light fractional ablation 87, 88, ultrasound application 89–91, sandpaper grinding 92, microneedle rolling 93, 94 and mechanical compression 95–99.

**Figure 5:**
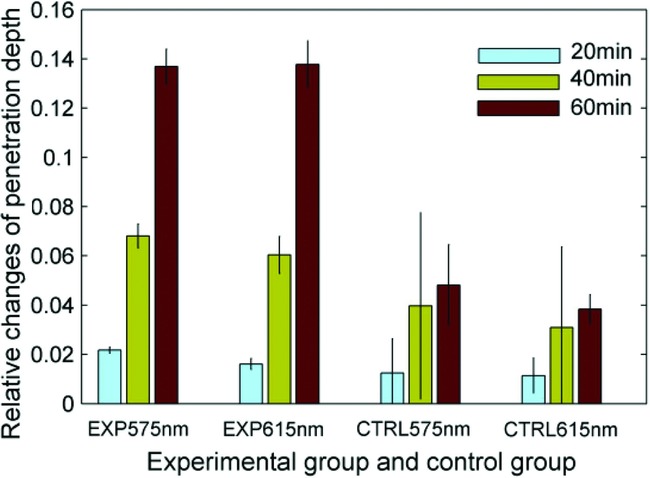
(online color at: http://www.lpr-journal.org) Relative increase rate of the light penetration depth for Q-switched Nd:YAG laser irradiation (EXP) and non-irradiated (CTRL) skin samples after glycerol treatment for 20, 40 and 60 min 84.

Different light sources (i.e. CO_2_ laser, Nd:YAG laser operating at 532 and 1064 nm) with different doses, and different pulse modes (e.g. IPL sources operating at 400–700 and 560–950 nm) were used to irradiate skin in vivo prior to application of OCAs. The reflectance spectra of skin were measured before or after treatment of irradiation and OCAs, respectively. It was found that both Q-switched and long-pulsed Nd:YAG laser (1064 nm) irradiation could induce the optimal permeability for facilitating OCAs transepidermal delivery. The relative decrease of reflectance for skin samples with a combination of irradiation and topical application of OCAs was up to 8–9 times more than that of nonirradiated skin samples but topically treatment of OCAs alone 84. An in vitro study also demonstrated that Q-switched Nd:YAG laser irradiation at 1064 nm with 100 mJ pulses helped ti increase the light-penetration depth. The tissue absorption coefficient and reduced scattering coefficient were calculated based on measured reflectance and transmittance spectra with a spectrophotometer and an integrating sphere, as described above. The results clearly demonstrated that after 60 min of glycerol treatment, the penetration depth increases up to 5 times compared with that of the control group that only received glycerol treatment without irradiation (see Fig. 5).

**Figure 6:**
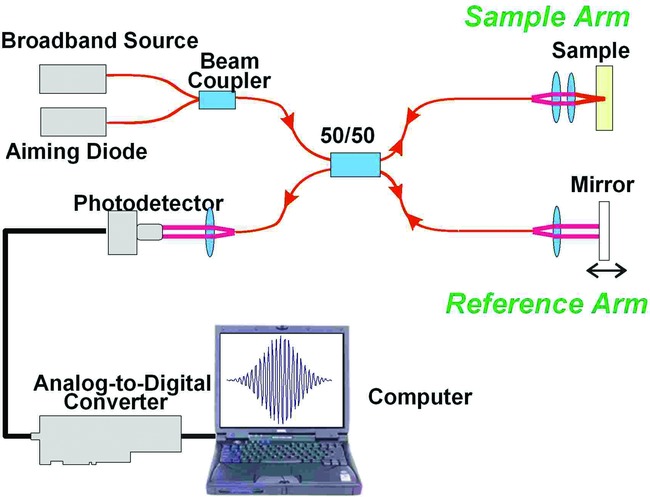
(online color at: http://www.lpr-journal.org) Simplified schematic diagram of a typical first-generation (time domain) OCT system. OCT is a relatively new noninvasive optical diagnostic technique that provides depth-resolved images of tissue with resolution of a few micrometers at depths reaching a few mm in highly scattering tissues such skin and sclera of the eye 115–118. The basic principle of OCT is to detect backscattered photons from tissue within a coherence length of the source by using a two-beam interferometer. In its basic operation, the light back-reflected from the tissue is combined with light returned from the mirror in reference arm of an interferometer (Michelson or Mach-Zehnder), and a photodiode detects the resulting interferometric signal. Interferometric signals can be formed only when the optical path length in the sample arm matches the reference arm length within coherence length of the source. Therefore, as low-coherence (broadband) laser source are used as the source on OCT systems, the fringes can be obtained from different depth location by modulating the position of the reference arm. By gathering interference data at points across the surface, cross-sectional 2-D and 3-D images can be formed.

In another study, Stumpp et al. 85 used a 980-nm diode laser to irradiate hamster and rat skin with added artificial absorption substrates on the surface. The laser irradiation was strongly absorbed and provided precise heating to breach the SC barrier. After successful removal of the absorption substrates, glycerol was applied to the skin surface. Optical coherence tomography (OCT, Fig. 6) was used to image the treated area to record the optical tissue properties for 30–45 min. The results indicated that there was an improvement of up to 42% in light penetration into the skin.

A flashlamp (IPL, 650–1200 nm, 525–1200 nm, 470–1400nm) system with the help of an island damage mask was also used to destruct the skin integrity 86. The mask with a pattern of carbon-black absorbing centers was applied to the skin surface to absorb light and to create lattice of islets of damage. Fresh rat skin, farm pig, and Yucatan pig skin ex vivo, and human skin samples ex vivo and in vivo were used to verify the optical-clearing efficacy of glycerol at different concentration, 40%-glucose as well as 60%-propylene glycol. After 1–1.5 h of OCAs application, a lattice of islands of limited thermal damage produced on the skin surface allowed for substantially enhanced penetration rate of topically applied OCAs, and enhanced the overall optical transmittance. The fractional laser microablation of the skin surface by 2940-nm erbium laser pulses with energies from 0.5 to 3.0 J (Palomar Lux2940, Palomar Medical Technologies Inc., USA) and the ultrasonic treatment may have also a good impact of OCAs delivery, including agent delivering by micro-nanoparticle containers 87, 88.

**Figure 7:**
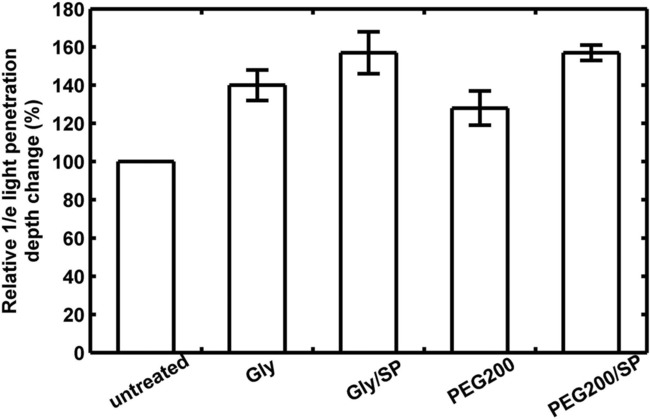
Changes in OCT imaging depth into in vivo optically cleared porcine skin with 60% glycerol, glycerol/, 60% PEG200, PEG200/at 1310 nm 89.

Xu et al. 89 used ultrasound with a frequency of 1 MHz to enhance OCAs permeation into skin, and the imaging depth of in vitro porcine skin was measured with OCT. Figure 7 summarizes the relative light-penetration depth value for 60% glycerol (Gly), 60% PEG200, and the combination treatment of sonophoretic delivery (SP) with Gyl and PEG200/SP at 60 min measured with OCT. It can be seen that the light-penetration depth increases 40%, 29%, 56%, and 56%, respectively, compared with the initial value. The ultrasound treatment produces a 40% and 93% enhancement over Gly and PEG200 alone treatment.

Abrasion of the skin SC by gentle rubbing with a piece of sandpaper also enabled enhancement of OCAs penetration and improve optical-clearing efficacy. A piece of fine 220-grit sandpaper was used by Stumpp et al. to gently rub glycerol or dextrose solution into hamster skin after hair depilation in vivo 92. The manual rubbing with OCAs on the skin surface was continued for two to four minutes in different directions and performed gently to avoid injury to the skin. A few minutes after the rubbing, the skin gradually became optical cleared, allowing visualization of subcutaneous blood vessels. The quantitative analysis of OCT signal indicates that the 1/e light-penetration depth was increased by 36–43%. The optical clearing can also be reversed by phosphate-buffered saline solution application.

The integrity of skin SC can also be directly breached by a mechanical tool such as a microneedle roller. Yoon et al. utilized it to enhance the penetration of glycerol into ex vivo porcine skin samples 93. Rolling for 50 times each in horizontal, vertical, and diagonal directions over the skin epidermis with an array of 192 needles (70 µm diameter and 500 µm height) on a cylindrical surface resulted in formation of microholes that served as many transdermal microchannels for OCAs permeation. All the skin samples were placed over a customized modulation transfer function target (MTFT) to acquire crosspolarized images, and found that relative contrast of the MTFT was increased approximately two-fold for the microneedle-roller-treated samples compared with the control samples 30 min after glycerol application. Combining ultrasound with microneedle-roller treatment of ex vivo porcine skin samples and then applying 70%-glycerol, the glycerol transepidermal diffusion rate of skin samples treated was approximately 2.3-fold higher than that treated by microneedle roller alone 94.

Tape stripping could also be performed to breach SC. Gentle application of about 30 strippings makes the skin surface glisten but not damaged. In addition, medical grade cyanoacrylate adhesive bonded on the slide surface could also be used to remove the SC 95. The slide with cyanoacrylate was applied to skin with moderate pressure for appropriate 3 min, then released and remained on the skin for an additional 2 min. When the slide was peeled, the SC was successfully adhered to the substrate and removed from skin. This process was repeated for 3–6 times to make the skin glisten. OCAs were subsequently applied to the treated skin area by a low-pressure transdermal application device, which also enhanced the OCAs permeation. Tattoos in the skin dermis with removed SC were much clearer seen than those with nonremoved SC 95.

A mechanical facility called TOC devices (TOCD) was first introduced by Rylander and coworkers 96, 97, which consisted of an array of pins fixed on the inner chamber surface and two brims to interface with the skin surface. When the device was connected to a vacuum pump (∼750 mm Hg), the brims formed an airtight seal and the pins induced spatially localized tissue compression. Using a hemispherically tipped glass rod 20 mm long with a 3-mm tip diameter as a probe for localized skin compression, it can be found that similar effective tissue strain, air dehydration and mechanical compression produce similar changes in refractive index and water-volume fraction. The mechanical compression can cause local water removal within compressed regions of tissue, i.e. the localized mechanical compression of skin decreases tissue thickness and water content and increases refractive index and OCT signal intensity 96, 97. Due to different mechanical properties and water content of epithelial and stromal layers, the compression-induced changes in scattering properties of these two layers are different. Thus, better contrasting of these layers in OCT images was demonstrated for human rectum ex vivo 98 and human skin in vivo 99. The temporal dependences of OCT image contrast for the epidermis–dermis junction measured at low and high pressures for volunteers of different ages were associated with different dynamics of water inflow, in particular connected with the different balance of free and bonded water for the young and aged skin and different elasticity of the skin of the various age groups 99.

### 3.2. Chemical-penetration enhancers

Chemical-penetration enhancers used in medicine and cosmetics were also applied for TOC. Azone 100, Oleic acid 101, DMSO 102–105, propylene glycol (PG) 106, and Thiazone 106–109 were shown to accelerate permeability of OCAs into skin to improve the skin optical-clearing efficacy.

For instance, 60 min after in vitro skin treatment by PG-Azone or glycerol-Azone mixture, the light transmittance at 1276 nm was increased by 37.3 and 41.1%, respectively; diffuse reflectance at 1066 nm was decreased by 20.6 and 29.3%, respectively. Compared with 80%-glycerol, the mixture of 40%-glycerol-Azone or 40%-PG-Azone could induce analogous optical clearing of skin, which indicated that the permeation of OCAs was markedly enhanced by application of the drug 100.

Oleic acid (OA) is a monounsaturated fatty acid and is widely used as a safe transdermal enhancer in the field of drug delivery. In skin optical clearing, the synergistic effect of OA as a promoter to facilitate OCAs to permeate into skin tissue has been studied in Ref. 101. DMSO was also used not only as a hyperosmotic agent to induce skin optical-clearing efficacy but also as a penetration enhancer. Previous studies have shown that the mixed solution of glycerol and DMSO was able to have a synergistic effect on optical clearing of gastric and skin tissue due to its carrier effect 102–105. However, there has been a common assumption about DMSO, which is that its toxicity profile is unacceptable for clinical use. Actually, DMSO is FDA-approved for treatment of interstitial cystitis, at a concentration of 50%, and the side effect of DMSO just involves the garlic odor that occurs with DMSO absorption by the body, and the irritation that is on a par with other commons agent, such as oleic and linoleic acids 119.

**Figure 8:**
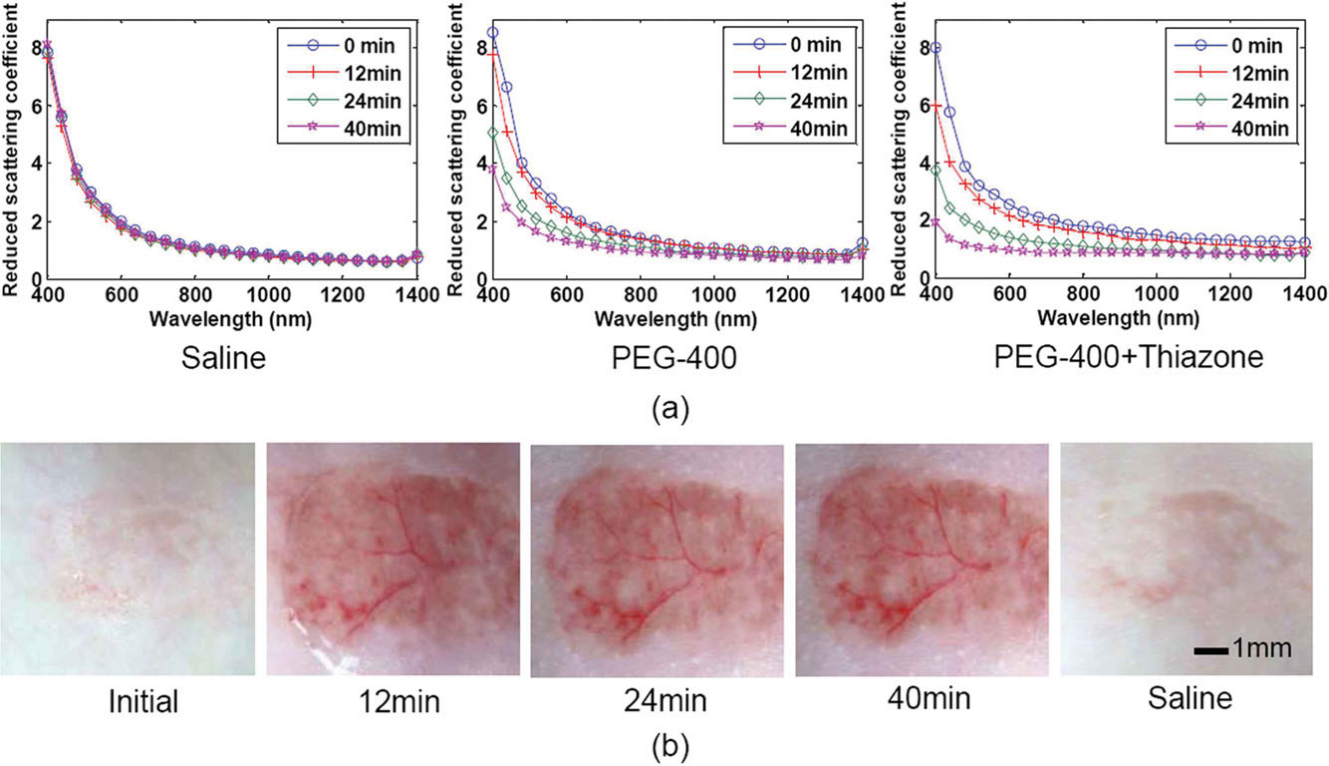
(online color at: http://www.lpr-journal.org) (a) Typical spectra of reduced scattering spectra of in vitro skin sample at initial state and different times (12, 24, and 40 min after the application of the chemical agents (saline, PEG-400, and a mixture of PEG-400 and Thiazone); (b) visibility of subcutaneous blood vessels of in vivo rat skin before and after treatment with (a) PEG400 and (b) PEG400-Thiazone mixture 33.

Thiazone, as an innovative penetration enhancer, has a skin-penetration effect ∼3 times of that of Azone, which was first introduced into the investigation of skin optical clearing by Mao et al. 107. Based on measurements of reflectance and transmittance spectra with a commercially available spectrophotometer (Lambda 950, PerkinElmer, USA) with an integrating sphere, the reduced scattering spectra of rat skin in vitro before or after topical treatment of saline, PEG-400 or the mixture of PEG-400 and Thiazone were calculated with IAD program, respectively (see Fig. 8a). It can be seen that the reduced scattering coefficient spectra decrease with treatment time, and the thiazone can induce the largest changes 33. For in vivo topically application, Thiazone also reveals the obvious ability to enhance OCAs to permeate into skin and makes the subcutaneous blood vessels clearly visible after treatment for 12 min, and recover after treatment of saline, which can be seen in Fig. 8b 33. In addition, comparing with Azone or PG, Thiazone possessed the highest penetration-enhancing effect for in vitro 106 or in vivo skin 108.

### 3.3. Combination of physical and chemical enhancement

As discussed above, a solo physical breaching or chemical enhancer was quite effective to induce skin optical-clearing efficacy. Sodium lauryl sulfate (SLS) is a surfactant and often used as a penetration enhancer in pharmaceutical and cosmetic products. The combined utilization (surgeon-performed [SP]) of ultrasound and SLS was performed on in vitro porcine skin and the synergistic enhanced effect on OCAs permeation was investigated in 109. After the solutions (60%-glycerol (Gly), Gly/SLS) were respectively applied to skin samples, ultrasound at 0.75 W, the combination of SP and SLS treatment exhibited the most effective enhancement in increasing light transmittance and improving OCT imaging depth, and reduced the treated time compared with SP solo treatment.

**Figure 9:**
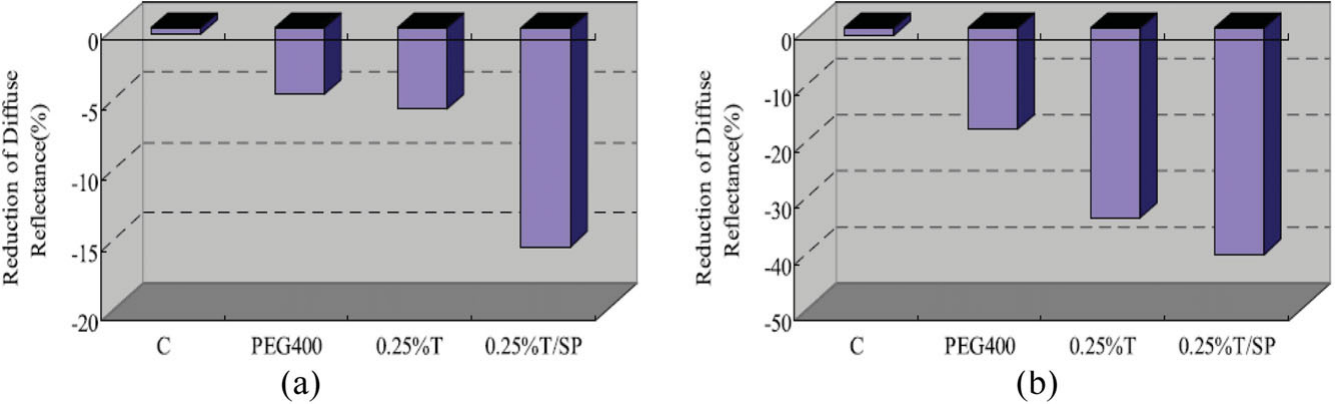
(online color at: http://www.lpr-journal.org) Reduction in diffuse reflectance at 540 nm for human skin after 10 min (a) and 60 min (b) treatment with nothing (C), pure PEG400, 0.25%-Thiazone (T), and 0.25%-Thiazone/SP (T/SP) 110.

Ultrasound treatment was also utilized to combine with Thiazone (T) to enhance PEG400 penetrate into skin 110. The mixed solution of PEG400/0.25%-T was applied to the surface of in vitro porcine skin samples and in vivo human skin. As is shown in Fig. 9, the diffuse reflectance decreased by approximately 33.7-, 3.3- and 2.7-fold in T/SP compared with that of the samples in Control, PEG400 and T, respectively. At 60 min, the decrease in diffuse reflectance of samples in T/SP is about 2.2- and 1.2-fold compared with that of the samples in PEG400 and Thiazone, respectively, at 540 nm. Also, the OCT imaging depth increased 41.3%, which was significantly different from the control samples.

The associative action of tape stripping with chemical enhancers, such as Thiazone, Azone and PG, has also been studied for rat dorsal skin in vivo 108. Tape stripping was implemented onto the epithelium surface for about three to four times to loosen the skin SC and then a mixed solution of enhancer/PEG400 was applied to the stripped skin area. As a result, the diffuse reflectance was decreased. The changes caused by the four kinds of agents vary, the largest changes in reflectance induced by PEG-400/Thiazone, and then, PEG-400/Azone second, PEG-400/PG third, and pure PEG-400, which is different from the results in vitro because in vitro porcine optical clearing experiments show that the penetration enhancing ability of PG was much better compared to Azone, and suboptimal to Thiazone 106.

## 4. Slicing tissue with optical clearing technique

### 4.1. Tissue optical clearing technique for improving imaging depth of microscopy

Traditionally, the cedar-wood oil or mineral oil has been used in classical microscopy to match the refractive indices of the sample and objective lens in order to improve the imaging quality due to elimination of random light scattering from tissue/cover slip/lens interfaces. However, the tremendous potential application of this technology for the enhancement of imaging depth and contrast of diffuse optical spectroscopy and microscopy was not regarded until Tuchin et al. proposed in 1996/1997 to control tissue optical properties using immersion agents by impregnating tissue under study by these agents 8, 9. For the former, the agent does not change the sample itself, but for the latter decreases the scattering coefficient because OCAs with higher refractive index penetrate into the tissue and partly replace tissue interstitial fluid.

**Figure 10:**
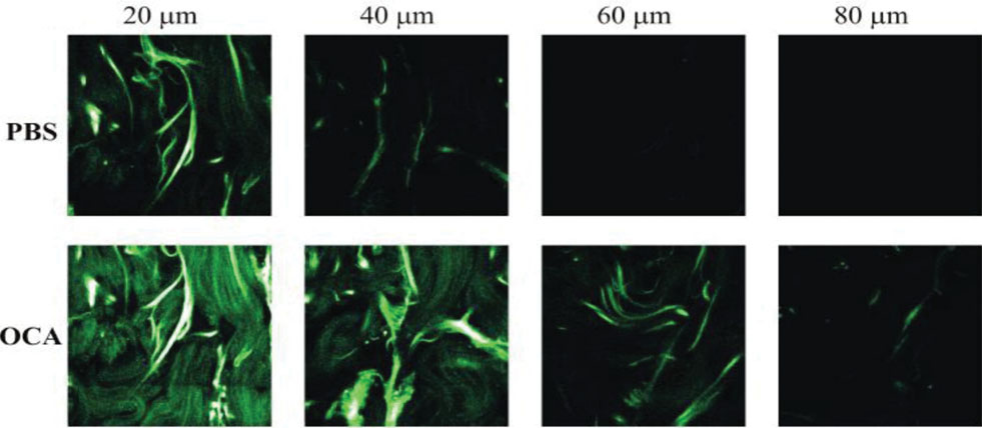
(online color at: http://www.lpr-journal.org) Images of ex vivo human skin tissue sample immersed in PBS (upper) and after immersion in glycerol (lower) for 7 min obtained with two-photon microscopy 43.

Typically, a standard laser-scanning confocal microscopy (LSCM) penetrates only to a depth of 100 µm below the brain surface, and the two-photon excitation fluorescence microscopy (TPEFM) can offer improved depth 2. In fact, the TPEFM imaging depth for skin is much less, which is limited to 40 µm depth. With TOC technique optical clearing, Cicchi et al. first reported double TPEFM imaging depth 43. The system scheme of TPEFM is similar to shown in Fig. 4c, but different components were used. A mode-locked Ti:sapphire laser (Coherent MIRA900) provides the excitation light, which comprises 100-fs width pulses at an 80-MHz repetition rate, tunable in wavelength between 700 and 1000 nm. The fluorescence is collected by the objective and retraces the same optical path as the laser excitation, encountering the two galvomirrors (descanning mode), before being separated out by a dichroic mirror and detected by an avalanche photodiode (Perkin Elmer SPCM-AQR). Figure 10 shows two typical image stacks; the upper row acquired with the sample immersed in PBS and the next row acquired 7 min after the sample was immersed in 0.5 ml of glycerol. The results indicate that the image contrast is enhanced and the signal is clearly improved for the same depth after application of glycerol, as well as the imaging depth increases from 40 µm to 80 µm. This study provides an option for the applications of two-photon microscopy or other optical biopsies for deep-tissue imaging.

### 4.2. Three-dimensional reconstruction of tissue microstructures

Since the TOC technique could significantly improve the penetration depth and image contrast of optical microscopy, investigators tried to combine these methods with fluorescent microscopy for the 3D reconstruction of tissue microstructures 44–54.

Chiang's group introduced a kind of OCAs called FocusClear 120. By direct immersion of a 500-µm tissue section in the solution, the biological structures, including animal and plant cells and organisms can become transparent. Combined with fluorescence label and LSCM, it allows one to visualize the microstructure and vascular network with subcellular-level resolution 48, 49. The optical sections at different depths of a typical islet are shown in Figs. 1a–e. Figures 1f and g are stereo projections of the blood vessels obtained by fluorescence imaging, and Figs. 1h and j show the corresponding 3D top view and bottom view of the islet vasculature created by using the Amira software to merge the signals. Figures 1j and k are two additional results of the high-resolution, 3D vasculature imaging of the mouse pancreatic islets 49. Therefore, the optical-clearing method was able to yield continuous and integrating optical sections and avoid the loss of information caused by making physical sections.

**Figure 11:**
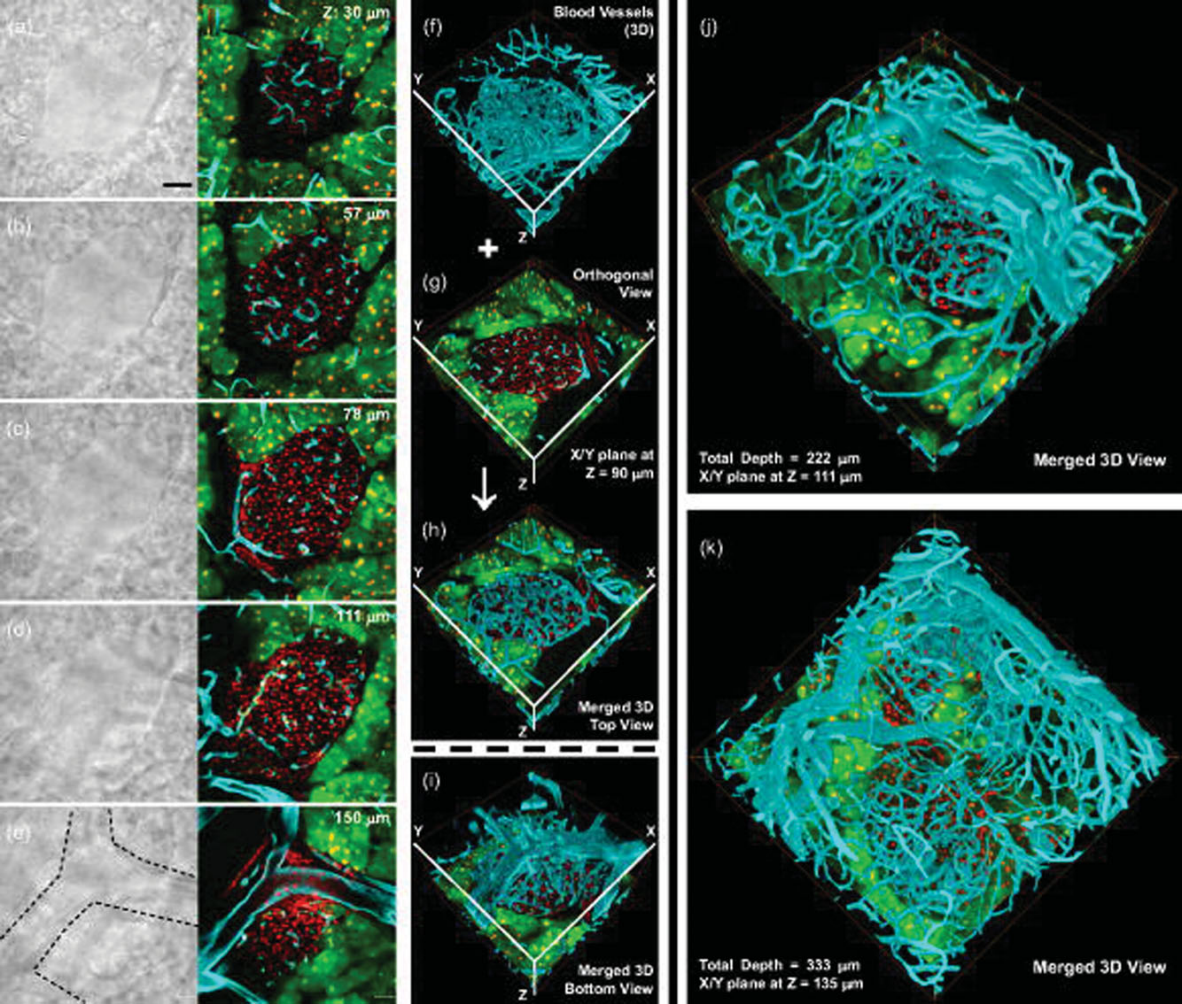
(online color at: http://www.lpr-journal.org) Three-dimensional images of islet vascular in the mouse pancreas. (a)–(e) Images of transmitted light (left) and fluorescence (right) at different depths in the pancreatic tissue specimen (vascular: cyan; nuclei: red). (f)–(i) Separate and merged stereo images of blood vessels and the islet/pancreatic structure. (h) and (i) Projections from the top and bottom halves of the imaged area. Dimensions of the scanned volume: 369 µm (x) × 369 µm (y) × 225 µm (z, depth). (j) and (k) Two typical three-dimensional projections of the islet vascular network 49.

Dickie and coworkers also developed a method for 3D in situ visualization of microvasculature of whole-mount tissue 44. After labeling cells and microvascular, the thick tissue samples, i.e. brain, adipose tissue, skeletal muscle obtained from adult mice were then cleared through running a series of dehydration and clearing treatment. They were able to visualize the microvasculature of various normal and tumor tissue samples to a depth of up to 1500 µm within a 3D context by LSCM.

### 4.3. Optical clearing technique for 3D reconstructions of neuronal networks

Neuroscience research has been a hot topic in life sciences. Techniques like computer tomography (CT) or magnetic resonance imaging (MRI) do not yield cellular resolution, and mechanical slicing procedures not only lose some important information, but also make the imaging process more difficult. Micro-optical sectional tomography (MOST) can be used to obtain a high-resolution atlas of the mouse brain, which provides a good tool for exploring the microstructure and function of neuronal networks, but it needs more than ten days to get an ultra-microstructure of whole mouse brain 121. Nevertheless, it is useful for light-microscopy-based connectomics of cellular networks in brain and other tissues, which is expected to significantly contribute to the discovery of new biological principles in connectomics and circuit genetics.

After the paraformaldehyde-fixed and labeled fly brains were cleared in the FocusClear solution for about 5 min, the brain samples were immersed in a drop of MountClear (CelExplorer, Taiwan) for confocal imaging, and the map of olfactory representation in the drosophila mushroom body was obtained. It was found that the olfactory coding at the antenna lobe (AL) was likely decoded in the mushroom body and then transferred via distinct lobes to separate higher brain centers 50. Without doubt, the combination of optical clearing and optical microscopy would significantly contribute to the studies of neuroscience.

**Figure 12:**
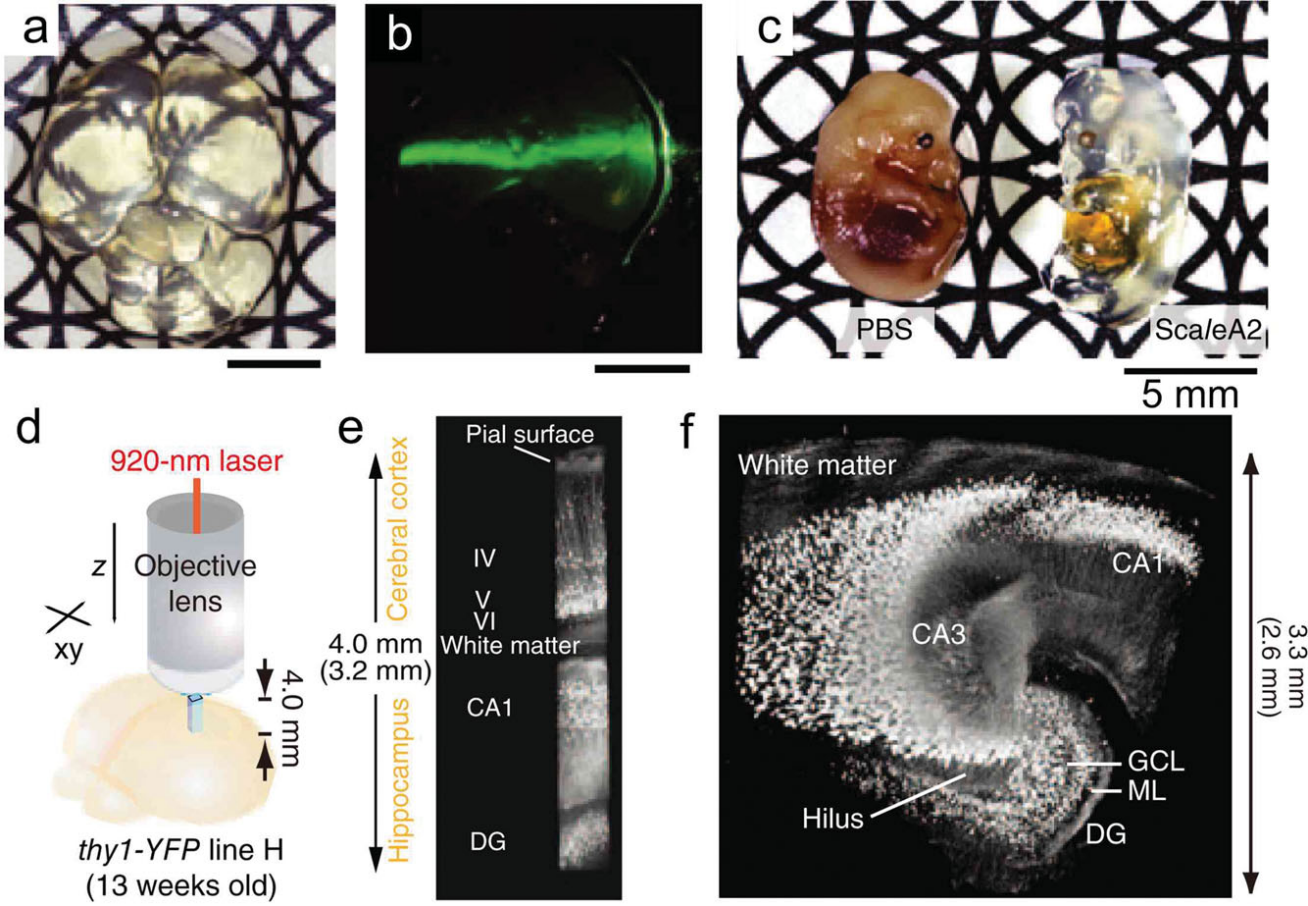
(online color at: http://www.lpr-journal.org) Tissue clearing performance of ScaleA2. (a) Cleared brain was taken with patterned background. (b) A 1-mW, 532-nm laser beam irradiated at the cleared brain. (c) Two embryos placed in PBS or incubated in ScaleA2 solution for 2 weeks after fixation, (d) TPEFM imaging using a custom-designed objective with a distance of 4.0 mm. (e) Three-dimensional reconstructions of YFP-expressing neurons in a quadratic prism located in the cerebral cortex and (f) hippocampus (m) and in 24 (4 × 6) quadratic prisms located in the excised hippocampus are shown. All scale bars represent 50 mm 51.

However, the imaging depth of 500–1500 µm is not enough to complete 3D reconstructions of neuronal networks of whole mouse brain. Hama et al. developed a clearing reagent called Scale that could not only make biological sample transparent, but preserve fluorescent signals in the cleared structures 51. After intact fixed mouse brain was immersed in the most effective solution named ScaleA2 for more than 2 weeks, the brain became very clear, as shown in Fig. 12a, and a 532-nm laser beam could pass through the brain as shown in Fig. 12b. Even whole mouse embryos can be effectively cleared as shown in Fig. 12c. In order to image deeper tissue with high resolution, the authors used a customized 25× objective lens, which has a long working distance (4.0 mm) and a sufficiently high NA (1.0). With this lens, it is possible to generate very long quadratic prisms of the YFP-H line brain, with reconstructions that extended from the brain surface to the dentate gyrus. Figure 12d shows the diagram for TPEFM imaging using this objective lens. Three-dimensional reconstructions of YFP-expressing neurons in a quadratic prism located in the cerebral cortex and hippocampus (Fig. 12e) and in 24 (4 × 6) quadratic prisms located in the excised hippocampus (Fig. 12f) are clearly visible 51.

**Figure 13:**
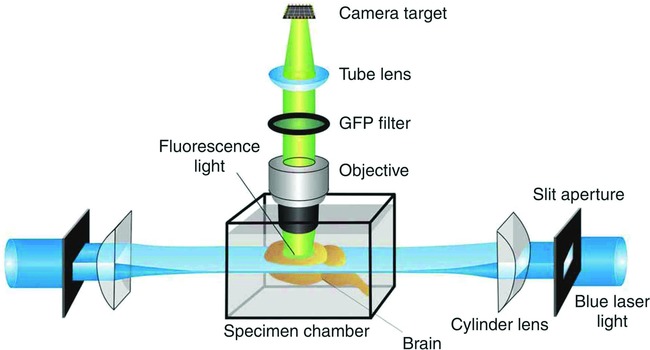
(online color at: http://www.lpr-journal.org) Schematic diagram of ultramicroscopy setup showing tissue positioning and the light path 52.

Dodt et al. used the idea of light-sheet illumination to develop an ultramicroscopy that allows one to observe macroscopic specimens like whole brains with microscopic resolution 52. Figure 13 show the diagram of a typical system. The specimen is illuminated from two sides by a blue laser forming a thin sheet of light, and fluorescent light is thus emitted only from a thin optical section and collected by the objective lens. Stray light is blocked by a filter and the image is projected through the tube lens onto the camera target. As all parts of the specimen above or below the light sheet are in the dark, no out-of-focus light is generated, and no light has to be excluded later, as in confocal microscopy. Comparing with confocal or two-photon microscopy, the ultramicroscopy can quickly produce good optical sectioning in macroscopic specimens because low-power objectives with a low numerical aperture (NA) can provide a large field of view. However, the application of ultramicroscopy completely depends on optically transparent objects, so they further developed new optical-clearing methods 42, 122, which push the development of the TOC technique.

**Figure 14:**
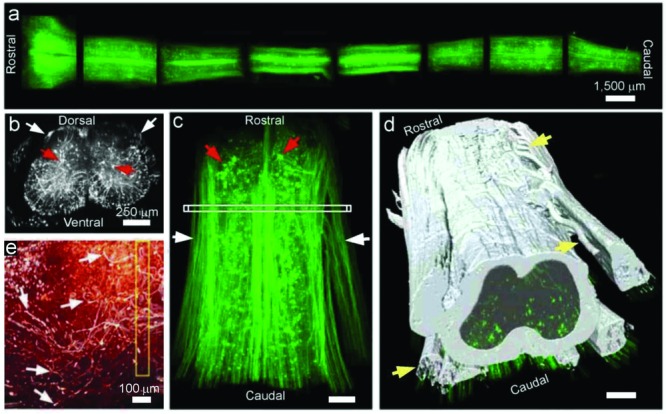
(online color at: http://www.lpr-journal.org) Imaging large regions of the spinal cord of GFP-M mice. (a) Entire spinal cord of a GFP-M mouse cut into 3- to 4-mm-long segments, cleared and imaged with ultramicroscopy; (b) Cross-view projection of the indicated region in c; (c) A spinal cord segment (length 4 mm) of a GFP-M mouse scanned with ultramicroscopy shown in a horizontal view; (d). Traced white and gray matter boundaries and axon bundles; (e) 3D reconstruction in sagittal views for gegeneration of conditioned axons in GFP-M mice imaged by ultramicroscopy 10 d after injury and subsequent clearing 53.

The TOC procedure mainly includes two steps, one is dehydration, and the other is clearing with high refractive index. They initially used alcohol and benzyl alcohol–benzyl benzoate (BABB) for tissue clearing, which worked well only for small tissues with strong fluorescence expression and did not work on adult central nervous system (CNS) tissue 52. Later, they screened for a substrate from about 10000 chemicals and found that tetrahydrofuran (THF)—instead of alcohol—in combination with BABB, could both fully clear the adult spinal cord and preserve its fluorescent signal 53. Using this method, they imaged 3- to 4-mm long spinal cord segments shown in Fig. 14a, and axons were visualized up to 4 mm (Fig. 14c). Figure 14b show the crossview projection (50-µm thickness) of the indicated region in Fig. 14c, which was virtually indistinguishable from histological sections. On the basis of the collected images, it was possible to trace the white and gray matter boundaries and axon bundles (Fig. 14d). Furthermore, they determined the trajectories of regenerating sensory axons after injury and found that growth-competent axons as well as axons that have been considered to be growth incompetent show regenerative features (Fig. 14e). The results suggest that visualizing cells in unsectioned CNS tissue holds promise for assessing experimental therapies in spinal cord injury and other neurological pathologies 52.

**Figure 15:**
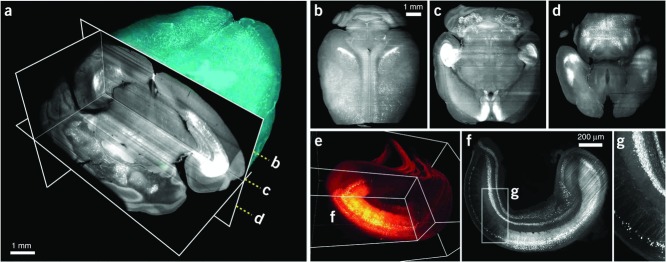
(online color at: http://www.lpr-journal.org) Typical results obtained from 3DISCO of neurons in the brain and hippocampus. (a) 3D reconstruction of an entire mouse brain with a corner-cut view; (b–d) The horizontal projections from the cleared brain at different depths marked in a. (e) 3D reconstruction of a cleared hippocampus with a corner-cut view showing some of the neurons that reside in the tissue; (f) The horizontal projection from the cleared hippocampus at the indicated plane in e. (g) Higher magnification of the region indicated in f 54.

Recently, Erturk et al. 54 also developed a new clearing protocol using dibenzyl ether (DBE) instead of BABB, in combina­tion with THF, and used ultramicroscopy to obtain 3D images of neurons in the whole mouse brain. This method is called three-dimensional imaging of solvent-cleared organs (3DISCO). In their study, the tissue clearing takes as little as 3 h, and imaging can be completed in ∼45 min. 3DISCO is a powerful technique that offers 3D histological views of tissues in a short amount of time. Figure 15a shows the 3D reconstruction of an entire mouse brain with a corner-cut view, and Figs. 15b–d demonstrate the horizontal projections from the cleared brain at different depths marked in Fig. 15a. The 3D reconstruction of a cleared hippocampus with a corner-cut view showing some of the neurons that reside in the tissue and their amplification of the region indicated in Figs. 15–g.

Therefore, the optical clearing technique solves the problem of low optical depth for imaging to a certain extent. Its significance was demonstrated in three-dimensional reconstructions of tissue and organ that contributed to the discovery of new biological principles in connectomics and circuit genetics. Combination of the optical clearing technique and various labeling techniques will bring great application prospects for life sciences in the future.

### 4.4. Depth-resolved assessment of molecular diffusion and clearing of tissues

Since the OCT technique has a unique capability of depth-resolved assessment of light distribution with high resolution, the change in the indepth distribution of the tissue scattering coefficient is reflected in change in the OCT signal. The slope of the OCT signal, plotted in logarithmic scale, is proportional to the total attenuation coefficient of tissues, *µ_t_*:


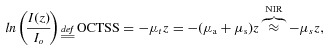
(1)

where *µ*_s_ and *µ*_a_ are the scattering and absorption coefficients, respectively (*µ*_s_ *>> µ*_a_ in the NIR spectral range).

In a simple model of scattering spheres, *µ*_s_ can be approximated as 123:


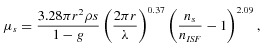
(2)

where *g* is the tissue anisotropy factor, *r* is the radius of scattering centers, *ρ*_s_ the volume density of the scatterers, *λ* the wavelength of the incident light, and *n*_s_ and *n*_ISF_ the refractive indices of the scatterers and surrounding medium, respectively. If the refractive index of the scatterers remains constant and is higher than the refractive index of the surrounding medium, the diffusion of molecules inside the medium reduces the refractive-index mismatch, Δ*n = n*_s_−*n*_ISF_, and, hence, the scattering coefficient is also reduced:


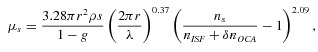
(3)

where *δn*_OCA_ is the OCAs-induced increase of *n*_ISF_. Therefore, an increase of tissue molecular concentration will raise the refractive index of the surrounding medium that will decrease the scattering coefficient. Thus, one can monitor and quantify the diffusion process by analyzing the changes in the OCT signal slope.

**Figure 16:**
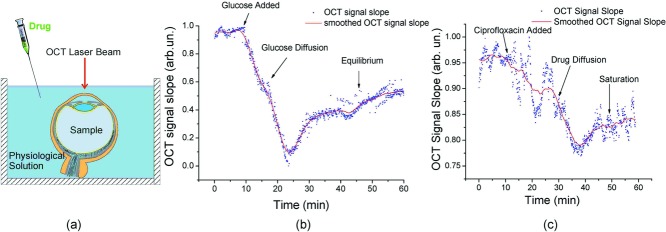
(online color at: http://www.lpr-journal.org) (a) Schematic diagram showing experimental protocol during in vitro experiments with cornea and sclera (laser beam configuration is shown for experiments with cornea). OCT signal slope as a function of time recorded from sclera during glucose (b) and ciprofloxacin (c) diffusion experiments 58.

Figure 16a shows the experimental protocol during in vitro experiments with cornea and sclera. The OCT signal slope as a function of time recorded from sclera during glucose and ciprofloxacin (an ophthalmic drug) diffusion experiment is shown in Figs. 16b and c, respectively 58. The OCT signal slopes as a function of glucose concentration were calculated from a 105-µm long region at a sclera depth of approximately 70 µm from the epithelial surface (Fig. 16b). The permeability coefficient in this experiment was measured to be 2.95 × 10^−5^ cm/s. Similarly, the calculated ciprofloxacin permeability coefficient was measured to be approximately 1.6 × 10^−5^ cm/s. These data clearly demonstrate the possibility of quantifying the molecular diffusion in tissues based on assessment of optical properties at specific depths.

**Figure 17:**
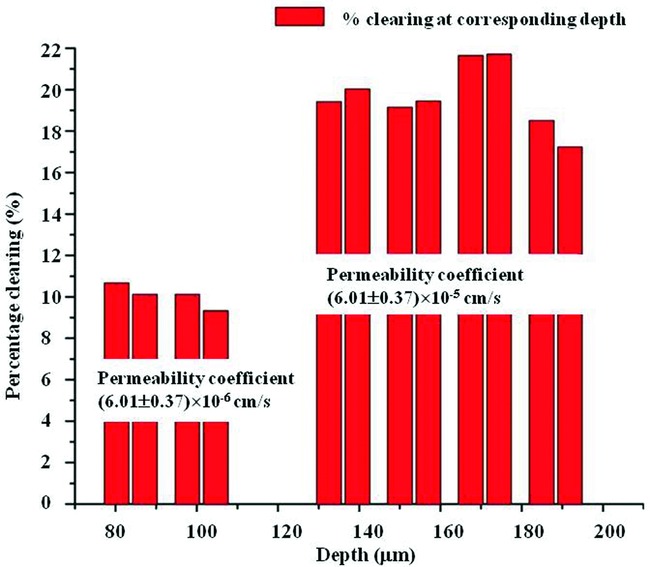
(online color at: http://www.lpr-journal.org) Optical clearing at different depth in a rabbit sclera during glucose 40% diffusion experiment 59.

In another work, Ghosn et al. assessed depth-resolved diffusion as well as depth-resolved clearing of glucose in the sclera 59. The permeability coefficients were calculated by analyzing OCAs-induced changes in OCT signal slope at different tissue depth while clearing – by monitoring change in OCT signal amplitude at those specific depths. Figure 17 demonstrates that the glucose permeability rate in regions in the epidermis region (upper 80–100 µm) is (6.01 ± 0.37) × 10^−6^ cm/s and significantly higher in the stromal region (2.84 ± 0.68) × 10^−5^ cm/s. In addition, optical clearing of the upper region was about 10% and increased to 17–22% in the stromal region. These results demonstrate the capability of the OCT-based method not only in measuring the diffusion rate and optical clearing of a tissue but also its ability of functional differentiation between layers of epithelial tissues.

As discussed in Ref. 9, human or animal sclera is a good model to study different clearing agents and drug-delivery pathways, because of its unique structure and optical properties, which relates it with bloodless skin dermis, dura mater and other connective tissues. The multilayer structure, including epithelial, stromal, and endothelial, makes it a good model to study OCAs diffusivity in these different layers. It is normally white with very reproducible reflectance and transmittance spectral properties and can serve as a natural white standard, like Intralipid. Besides, it is easy to collect from many animals, typically bovine, rabbit, rat, and porcine scleras, the structure and optical properties of which are very close to human sclera used in experiments. It is also easy to move from in vitro to ex vivo (whole eye ball) 124 and in vivo studies 125. Sclera as a tissue under study is also important for applications, such as transscleral photocoagulation of the ciliary body in laser therapy of glaucoma on optical clearing 124, 126.

It is interesting to note that longitudinal and depth-resolved analysis of tissue optical properties with OCT allows reconstruction of 2D diffusion maps. The visual representation of the molecular diffusion front was first demonstrated by Ghosn et al. while quantifying the permeability rate of glucose solutions in rhesus monkey skin noninvasively and in vivo 52. Recently, 2D visualization of transcutaneous delivery of a nanoparticulate peptide vaccine into mouse skin in vivo was also demonstrated in Ref. 65. Thus, OCT can provide a unique tool not only for the quantitative assessment of the OCAs and other molecules diffusion in real time and completely noninvasively but also visualize the diffusion front both in vitro and in vivo.

### 4.5. Molecular diffusion in normal and pathological tissues

**Figure 18:**
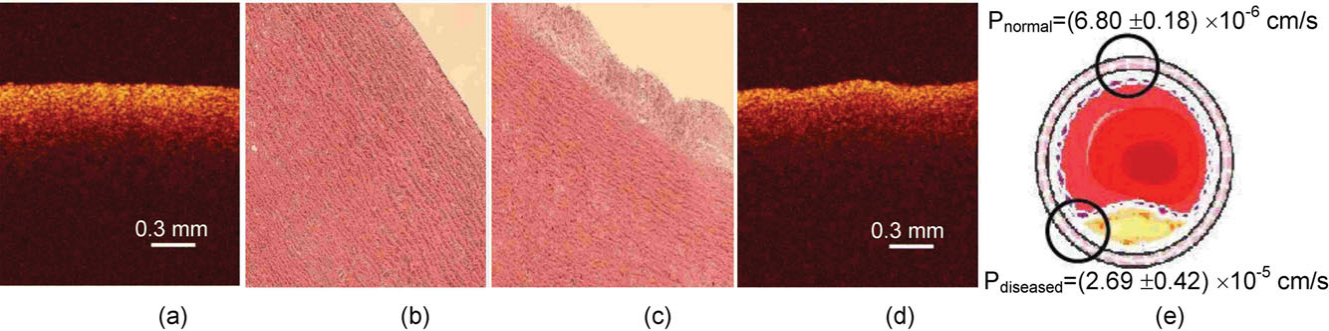
(online color at: http://www.lpr-journal.org) (a, c) OCT images of a normal and atherosclerotic porcine aorta respectively; (b, d) corresponding histology for the normal and atherosclerotic specimens; (e) permeability rate of glucose through atherosclerotic aorta compared to normal aorta 60.

An exciting new development came out from the above-described capability of OCT for depth-resolved assessment of molecular diffusion in various tissues. First, as noted by Ghosn et al., the permeability rates of selected OCAs were significantly different in normal and pathological vascular tissues, even before they could be assessed by any other imaging method (except invasive histopathological analysis) 60. They found that average permeability of 20%-glucose in normal tissues was (6.80 ± 0.18) × 10^−6^ cm/s (*n* = 4) while in arteriosclerotic tissues – (2.69 ± 0.42) × 10^−5^ cm/s (*n* = 7) as shown in Fig. 18e. The functional images provided by the OCT enabled effective distinguishing of normal and abnormal tissue. However, the structural OCT images from these experiments did not allow effective differentiation of normal from diseased tissues (Figs. 18a and c). This information could significantly increase the specificity and accuracy of tissue classification and further OCT's use in clinical diagnostic imaging.

**Figure 19:**
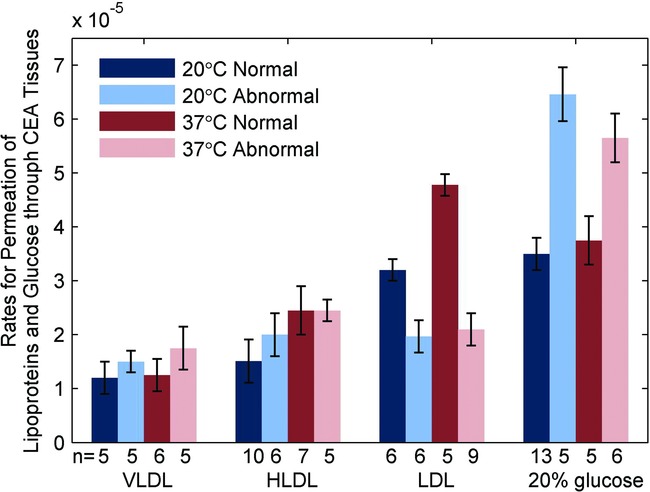
(online color at: http://www.lpr-journal.org) Comparative data acquired from lipoprotein perfusion experiments; Analyte type vs. Permeability Rate for VLDL, LDL, HDL, and glucose in both normal and diseased human carotid endarterectomy tissue samples, at 20°C and 37°C. Here n indicates the number of sample 62.

This method was further investigated to assess diffusion of other small and large biomolecules such as very low density lipoprotein (VLDL), low-density lipoprotein (LDL), and high-density lipoprotein (HDL) in normal and atherosclerotic human carotid endarterectomy tissues (CEA) 62. Most interestingly, while diffusion of glucose, VLDL and HDL were lower in normal versus atherosclerotic vasculature, the rate for LDL permeation through normal CEA tissue was (4.77 ± 0.48) × 10^−5^ cm/s, that is significantly greater (*p* < 0.05) than the rates for atherosclerotic CEA tissue (2.01 ± 0.23) × 10^−5^ cm/s (see Fig. 19). This was direct experimental support of a hypothesis that there is a facilitated diffusion of LDL molecules in vascular tissues.

Several further studies investigated the possibility of differentiation normal versus pathological tissues based on the molecular permeability rates in other tissues types such as breast 68, 69, esophagus 66, and lung 69. For example, it has been demonstrated that the permeability coefficients of 30%-glucose increase up to 32%, 113% and 162% in the benign granulomatosis, adenocarcinoma tumor, and squamous cell carcinoma of human lung tissue compared with that from the normal lung tissue, respectively 63. Moreover, change in the molecular permeability allowed classification of “aging” process of excised tissues 73. All of these results suggest that this method has a great potential for early identification of pathological alternations of the tissues and can be additional biomarker of tissue health.

It should be noticed that the molecular weight and form of OCAs could be an important characteristic of molecular diffusion in tissue. OCA diffusion through a biological membrane (tissue) in approximation of free diffusion is characterized by a diffusion coefficient 21:


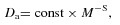
(4)

where *M* is the molecular weight of an agent, and the value of the parameter *S* depends on molecular form and size. The characteristic diffusion time (*τ*) through a tissue layer of thickness *d* for an OCA with the diffusion coefficient *D*_a_ is defined as


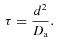
(5)

In general, parameter *S* for molecule diffusion in water is in the range 0.3–0.5, and for diffusion through a biological membrane it is about 3.5. For example, changing of the molecule weight *M* from 45 to 122 Da at diffusion in water changes the diffusion coefficient from 1.6 × 10^−5^ cm^2^ s^−1^ to 0.8 × 10^−5^ cm^2^ s^−1^ (2 folds) and for the same molecules diffusion through the plasmatic membrane − from 1.4 × 10^−8^ cm^2^ s^−1^ to 2.0 × 10^−10^ cm^2^ s^−1^ (70 folds). On topical application drugs penetrate into body by diffusion through a relatively thin epithelial cell layer, a thicker stromal layer, and then to blood vessels contained in the stromal layer. However, previous investigation shows that skin optical-clearing efficacy is not relative to the molecular weight of OCAs, which should be due to the small molecular weights of less than 400 D and relatively small role of molecule diffusion through thin cell layers. For a larger range of molecular weights, further investigations are needed to study the dependence of the optical-clearing efficacy on molecular weight.

## 5. In vivo application of TOC technique

The TOC technique is not only applied to in vitro imaging, but also shows enormous potential for in vivo optical imaging, diagnosis and therapy. Before the optical-clearing method is applied in vivo, it is necessary to investigate the safety of optical-clearing agents to these tissues, for example, whether there are short-term or long-term side effects on skin, blood vessel, organs or the body. In this section, the investigations on effects of OCAs on morphology of skin, blood vessels and blood flow are reviewed. Finally, the in vivo imaging and therapy will also be introduced.

### 5.1. Effects of optical-clearing agents on morphology of skin

Glycerol is one of the most common OCAs of skin, however, its optical-clearing efficacy is not very high during topical treatment of skin in vivo due to its relatively low penetration through SC. So, it is usually adopted dermal injection of glycerol 25. It has been noted that a high concentration of the glycerol can significantly alter the structure of biological tissue, even stop blood flow by obstructing the blood vessel 15; whereas low-concentration solutions do not have significant optical-clearing effect. Mao et al. 114 observed the dynamical changes in morphology of local skin during 2 weeks after dermal injection of 0.05 ml glycerol in different concentration, and they found that the glycerol solution with high concentrations (40%, 50%, and 75%) would lead to the necrosis of skin in the area of the application, but the skin was irreversible recovered after 2 weeks. Also, the glycerol solution with lower concentrations (20% and 30%) did not cause damage to skin. In practical applications, the optical-clearing agents are usually treated topically, and the doses of OCAs that penetrate into skin are very small, and the effects of optical-clearing agents on skin would be reduced.

Thiazone, a chemical-penetration enhancer, can facilitate the permeability of optical-clearing agents. Zhu et al. topically treated the mixture of PEG400 and Thiazone on the epidermis of rat skin, and monitored the dermal blood vessels and measured the blood flow 33. Further, they investigated the safety of skin after the topical application of the optical-clearing agents by observing skin morphology and microstructure. After topical application of the mixture for 30 min, the epidermis becomes thin, its thickness decreases from 59.36 ± 8.68 µm to 21.06 ± 2.31 µm 127. The SC was destroyed and the arrangement of subepidermal collagen became loose. Then, after a 48-h recovery, inflammation occurred inside the epidermis of skin, but the collagen arrangement become dense again. After 2 weeks, the skin began to rebuild, some vacuoles occurred in the epidermal layer of the skin for the superficial edema. After 4 weeks, the skin recovered to its initial state, and the regeneration of skin was not affected by the optical-clearing agent 127, 128.

In conclusion, topical application of optical-clearing agents may cause some degree of inflammation to skin in a short time, but the morphology and microstructure of skin can be recovered after several days. In the future study, the metabolic toxicity of optical-clearing agents to the organ systems of the body should be considered, and the body growth condition should also be investigated. More important is how to reduce the short-term side-effects of optical-clearing agents on in vivo application.

### 5.2. Effects of optical-clearing agents on blood vessels and blood flow

One of the primary goals for TOC is to allow for noninvasive visualization of the blood vessels and measure blood flow. Thus, apart from skin, it is necessary to investigate whether the action of optical-clearing agents would induce damage to the blood vessels themselves. For example, Vargas et al. investigated the effects of hyperosmotic agents on morphological changes of blood vessels 15. The glycerol solutions with different concentrations (100% and 75%) were directly applied on the subdermal side of hamster dorsal skin in vivo. They found that the application of glycerol induces occlusion of blood vessels; however, the morphological changes in blood vessels could be reversed by application of phosphate-buffered saline. Galanzha et al. also demonstrated that direct application of glycerol and glucose solution to microvessels of rat mesentery would induce short-term local stasis of the microvessels with some degree of dilation, but the side effect can be reduced by reducing the concentration of OCAs 25. Therefore, the challenges for the design of an optical-clearing method for in vivo application were not only for the clearing effects and the duration of the action of the chemical agents but also for the influence of the OCA on the tissue's normal physiological function.

**Figure 20:**
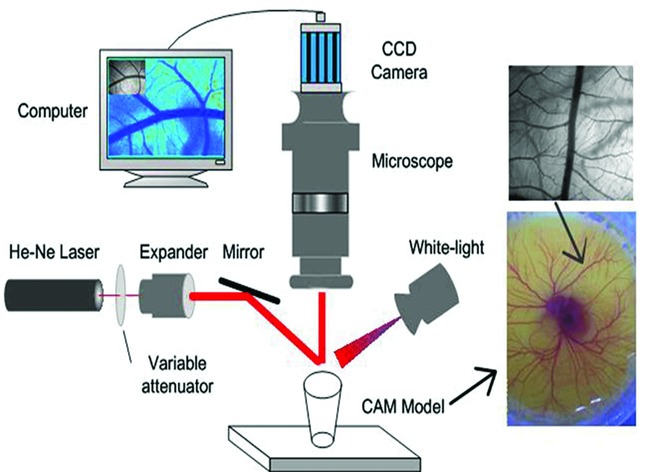
(online color at: http://www.lpr-journal.org) Schematic diagram of typical laser speckle contrast imaging system 129.

Cheng et al. used laser speckle contrast imaging (LSCI), a full-field blood flow imaging technique, to quantitatively evaluate OCAs-induced changes in blood flow 24. They found that after removing the rabbit skull and a small area of dura mater, the application of glycerol on the dura mater around the exposed cortex decreases the cerebral blood flow by 20–30% immediately. However, after a short time (∼70 s), the blood flow velocity would begin to increase and the dura mater become turbid again due to wash out of glycerol 24. Laser speckle is an interference pattern produced by light randomly reflected or scattered from different parts of an illuminated surface. When the scattered particles move, a time-varying speckle pattern will be generated at each pixel in the image. The spatial and the temporal intensity variations of this pattern contain the information about the scattering particles velocity. The LSCI technique gives the two-dimensional blood flow distribution with high spatial and temporal resolutions through analyzing the spatial blurring of the speckle image obtained by a CCD 24. Figure 20 shows the schematics of LSCI system. A 632.8-nm He-Ne laser (5 mW) was used to illuminate the area of interest with a beam expanded through a collimating lens. A sequence of raw speckle images was captured through a stereo microscope (SZ61TR, Olympus, Japan) by a CCD camera (Pixelfly USB, PCO Computer, Germany). When a white light is used instead of the laser source, the white-light image of the illuminated area can also be imaged by the CCD and recorded by the computer.

**Figure 21:**
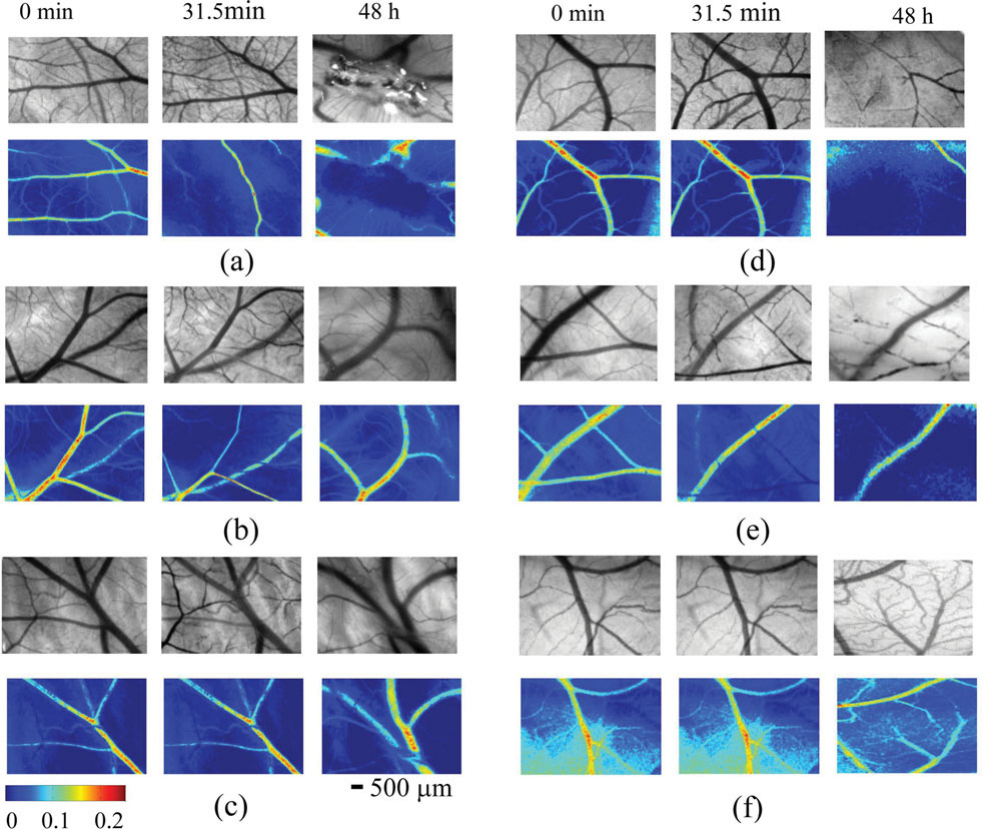
(online color at: http://www.lpr-journal.org) White-light (top row) and speckle velocity (bottom row) images before, 31.5 min after and 2 days after the application of different agents (40 µl): (a) 100%-glycerol, (b) 50%-glycerol, (c) 25%-glycerol, (d) 40%-glucose, (e) 20%-glucose, and (f) saline 129.

Zhu et al. 129 applied the LSCI to investigate both the short-term and long-term effects of glycerol and glucose on blood vessels in chick chorioallantoic membrane (CAM). Figure 21 shows the typical white-light images (upper) and blood velocity maps (below) before, 31.5 min later, and 2 days after application of 40 µL of glycerol, glucose at different concentrations or saline. The color bar indicates that the brighter the region, the faster the blood flows. Figure 21f shows the blood flow from control group 2 days after treatment by saline, in which only a part of the branch vessels in the same region can be seen because the diameter of blood vessels clearly increases and new blood vessels occur with the development of chicken embryo. It can be seen from Figs. 21a–e that direct application of OCAs would decrease the blood flow velocity or even block the blood vessels in a short time. Long-term observations indicate that the agents stunt the development of blood vessels in the CAMs. The short-term effects of glycerol are very strong and are confined to a local region, but the long-term effect is relatively light. The blood flow can be recovered to different extents if the blood vessel is not blocked completely. In contrast, long-term effects of glucose are more serious than short-term effects. The side effects depend on the dose of OCAs.

Mao et al. directly applied 30%-glycerol on the dermal blood vessels of rat skin flap window and laser speckle contrast imaging monitored changes in blood flow. The authors found that the blood flow velocity decreased initially and began to recover after 16 min. Thus, it can be concluded that the short-term effect on blood flow of the skin flap window can be revisable after the application of glycerol, but the longer-term effects should be further investigated 130.

### 5.3. High-resolution imaging dermal blood flow through the intact rat skin

Several noninvasive optical methods have been used to investigate skin microcirculation in clinical settings as well as improve disease management. For example, capillaroscopy allows the investigation of skin capillary morphology and density, laser Doppler flowometry allows real-time assessment of perfusion of skin volume, and transcutaneous measurement of oxygen tension provides continuous information about skin oxygenation 131, 132. Laser speckle contrast imaging can produce a two-dimensional map of blood flow with high spatial-temporal resolution 133–137, which plays an important role in studying of blood flow of various transparent tissues 137–141, such as, cerebral blood flow 138, 139, mesentery microcirculation 141, etc.

**Figure 22:**
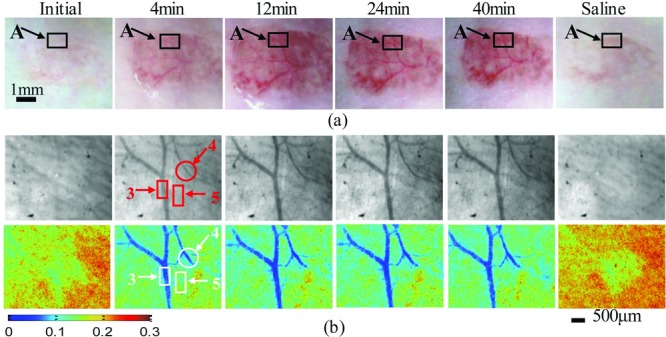
(online color at: http://www.lpr-journal.org) (a) Photographic images of rat skin in vivo before and after treatment with the mixture of PEG400 and Thiazone. (b) White-light (top row) and temporal contrast images (bottom row) of rectangle areas in (a) after treatment with the mixture of PEG400 and Thiazone 33.

Zhu et al. 33 developed an optical-clearing method to make the skin transparent and imaged the dermal blood flow through skin. Figure 22a show that large vessels through the skin can be seen after having topically applied a mixture of PEG400 and Thiazone to rat dorsal skin in vivo for 4 min, up to 12 min, the small branch vessels can be distinguished clearly. After 40-min treatment of the mixture, the saline solution was applied onto the area of interest. Immediately, the skin is recovered to the initial state and the blood vessels become invisible to the naked eye. Figure 22b shows the magnified white-light image (upper) and speckle contrast image (below) of the region in the rectangle presented in Fig. 22a. It can be seen that the image contrast becomes better after topical application of the mixture and more details of the dermal blood vessels or blood flow can be seen. The treatment by saline can make the contrast of the image decrease and the dermal vessels become invisible very quickly. Recently, they developed a switchable skin window by topical application of the mixture and saline on rat skin in vivo. With the repair effect of hyaluronic acid and Vaseline, it is able to repeatedly visualize the dermal blood vessels and flow distribution. Long-term observation shows that there is no abnormal reflection in microstructure, body weight, organ coefficients, histopathologic lesions, or toxic reactions compared with a control group. This switchable window will provide an effective tool not only for cutaneous microcirculation with laser speckle contrast imaging, but also for diagnosis and treatment of peripheral vascular diseases, including tumor research with various optical imaging techniques 127.

This optical-clearing method enables observation of dermal blood vessels and imaging of dermal blood flow through intact skin using the LSCI technique. Furthermore, by combining this method with the intrinsic optical imaging technique, it is possible to access the metabolic oxygen information of skin microcirculation. It is very important to diagnose peripheral vascular disease based on subcutaneous microcirculation. Also, with certain techniques, the pharmacological effect of vascular drugs can also be evaluated.

### 5.4. Skull optical clearing for imaging cortical blood flow

Optical imaging techniques have provided potential power to investigate cortical structure and function in vivo. For instance, LSCI has been widely applied to map the changes in cortical blood flow, which has provided valuable insight into many aspects of cortical function 138, 139, 141. Applications of TPEFM have contributed to our understanding of a broad array of neurobiological phenomena, including the dynamics of single channels in individual synapses and the functional organization of cortical maps 142. However, the high scattering of the skull becomes a barrier of optical neuroimaging in vivo, so craniotomy was applied to establish cranial windows 143–147, i.e. remove skull 138, 139, cranial window 143, 144, thinned-skull cranial window 145, 146, and polished and reinforced thinned skull window 144, etc., but the surgical operation changes the normal physiological environment of the cortex. Current investigations also give good perspectives for hard-tissue TOC, which were already demonstrated for tooth tissue 34, especially, cranial bone 53, 148.

**Figure 23:**
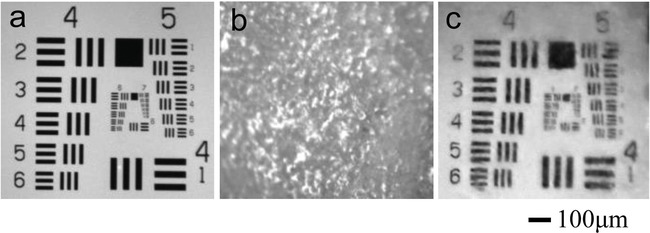
White-light images (a) of the central region of a 1951 USAF target; (b)–(c) images of the USAF target through the in vitro skull before and 25 min after treatment with SOCS, respectively 34.

Common optical-clearing agents of soft tissue, glycerol or propylene glycerol (PG) were applied to immerse the skull in vitro for 4–6 h, and showed a reduction of the scattering of the skull 142. Recently, an innovative skull optical clearing solution (SOCS) was invented to make mice skull transparent quickly 34. Figure 23 shows that the intact skull conceals the target completely; while the target is clearly visible through the treated skull by SOCS.

**Figure 24:**
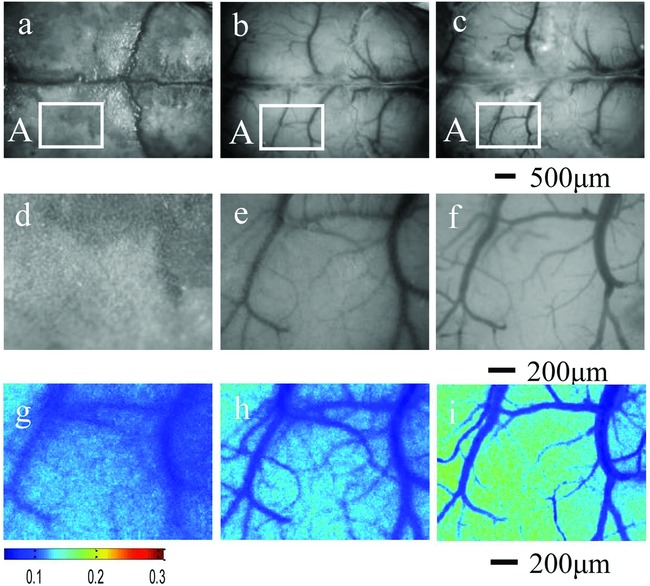
(online color at: http://www.lpr-journal.org) White-light images of intact skull (a), the transparent skull after SOCS treatment for 25 min (b) and remove rectangle area A (c). The corresponding magnified white images (d–f) and speckle contrast images and (g–i) of the rectangle area A shown in (a–c) 34.

These data demonstrate that the SOCS can produce an excellent optical-clearing efficacy for skull in vivo. Figures 24a–c, show typical white-light images of the intact skull, the treated skull and the removed skull rectangular area *A*, and Figs. 24d–f and g–i are the magnified white-light images and speckle contrast images of the rectangular area *A*, respectively. It can be seen that the intact skull is turbid, and the cortical blood vessels are hardly distinguishable. After treatment with SOCS, the skull becomes transparent, and the cortical microvessels can clearly be observed. Through the exposed cortex and the transparent skull after treatment with SOCS for 25 min, the minimum resolution diameter (MRD) of microvessel is 12.8 ± 0.9 µm and 14.4 ± 0.8 µm, respectively. The speckle contrast images show that the only blood flow of a few large vessels can be measured hazily through the intact skull, while the blood-flow distribution of cortical microvessels can be clearly distinguished in detail through the transparent skull, which is consistent with that through the exposed cortex. Therefore, it can be concluded that the skull optical-clearing method variously enhances the contrast of both white-light and speckle images, which provides an innovative transparent cranial window for LASC for accessing high-resolution cortical structural and functional information, and avoids the limitations of craniotomy-based cranial window with craniotomy.

### 5.5. Therapeutic applications of TOC

In light-based therapeutics, dermal scattering diminishes the depth of light penetration and attenuates the effective fluence. For laser tattoo removal, a higher radiant dose may be required to produce the desired therapeutic effect, particularly for more deeply located chromophores, but also with the increased risk of epidermal and dermal injury 23. Beneficially, at TOC the incident density of the laser energy can be reduced in dependence on the tattoo localization depth on 50–60% in blue-green, on 30–40% in red, and 10–20% in the NIR spectral range 23, 149–152.

Khan et al. 23 reported that the use of hyperosmotic optical-clearing agents, which are composed of a prepolymer mixture of polypropylene glycol and polyethylene glycol, PPG:PEG, enhances the laser penetration into intact human skin and temporarily alters the local optical properties in vivo. They topically applied the clearing agent (PPG:PEG) prior to Q-switched 532 nm and 694-nm laser treatment of human skin in vivo from a 40-year-old man (SPT IV) for a 15-year-old India ink arm tattoo. A Q-switched ruby laser (694 nm) was used at a fluence of 6 J/cm^2^ with a 4-mm diameter spot size. The results demonstrate that the combination of topically applied clearing agents (PPG:PEG) and laser is much more effective without damaging the epidermis. Prospective, comparative, and controlled clinical studies should be further performed so that the role of topically applied optical-clearing agents in light-based therapeutics can be fully understood.

## 6. Conclusion

This paper provides a review of recent developments and applications of the TOC technique. The physical mechanisms, molecular mechanisms, physiological mechanisms based on in vitro or in vivo tissue models are described that form the basis for developing safe and effective optical-clearing methods. In general, the mechanisms of TOC are usually due to multiple factors as follows: hyperosmotic agents induce dehydration of tissue, decrease the thickness of tissue; molecular dynamical reactions between tissue and OCAs cause dehydration of tissue or change tissue structure temporarily; OCAs increase the refractive index of interstitial fluid and enhance refractive-indices match among various substances in tissue. Since each tissue has its specific molecular composition and structure, different interaction between tissue and OCAs has been demonstrated that, not surprisingly, show different TOC processes. Tissue in vivo depends on physiological environment, so the mechanisms of tissue clearing in vivo are more complex and more difficult to describe. For future consideration, developing in vivo optical methods is needed in order to obtain both physiological parameters and optical properties, which will provide tools for investigation of mechanisms of optical clearing of tissue. Understanding the mechanisms of TOC is not only the basis of screening or designing OCAs, but also helpful for developing safe optical-clearing methods.

Optical-clearing efficacy of tissue is related to the local concentration of OCAs. Developed physical methods, chemical-penetration enhancers and combination of the both are playing an important role to increase TOC efficacy. In addition, new OCAs were invented for soft or hard TOC. Combining optical clearing of tissue, microscopical imaging and labeling techniques, the microvascular and microstructure of soft tissues in vitro can be obtained with subcellular resolution, which will help to explore the occurrence and development of disease. In particular, based on transgenic animals, combination of the TOC technique and advanced microscopy techniques, it is possible to describe nervous connection pattern by imaging 3D microstructure of the brain or central nervous system, which is very significant for revealing cognitive function and mechanisms of neurological disease.

The combination of optical clearing of tissue in vivo, such as skin or skull, and noninvasive optical imaging will provide effective tools for in vivo optical imaging and diagnosis, which show a powerful potential for investigation in life sciences. The TOC technique can reduce scattering and enhance the penetration of light in tissue, which is also beneficial for phototherapy, i.e. laser tattoo removal. Before the optical-clearing method is applied in vivo, the safety of optical-clearing agents have been carefully studied, which include the effects of optical-clearing agents on morphology, microstructure and function of tissue. In this review, the progress in TOC for in vivo imaging and phototherapy was also described.

Of course, the applications and the development of TOC technique is not limited to skin, brain and bone tissues, the optical clearing of mucosal and sclera tissues are also investigated 125. Mucosal tumor imaging 66, laser disruption of pathological abnormalities, and precise tissue dissection and removal of small abnormalities 124, 153 will be the wider application of TOC. The possibility to image even deeper structures of sentinel lymph nodes at the single-cell level, follow up photoacoustic detection and photothermal purging of metastasis targeted by nanoparticles, was recently demonstrated with the help of TOC 154. In developmental biology OCT holds great promise as a routine research tool for 3D analysis of mammalian embryos, especially with usage of biocompatible OCAs 36. In vivo optical clearing of newborn rat in propylene glycol solution was demonstrated with the aim to study vessel network using optical imaging 155. Another large field of TOC technology application is drug-delivery monitoring, including optical glucose sensing 10–12, 14, 156–158, which is based on drug or glucose metabolic impact on tissue optical properties. In addtion, the TOC technique has shown enhancement for detecoring autofluorescence 159, near-infrared fluorescence 160, 161 and Raman spectroscopy 162 or imaging bioluminescence 163, holographic 164, polarization signal 165, etc., which has not been mentioned due to the length of this review. However, TOC technique combines with various optical techniques will play a more important role in biomedical imaging, diagnosis.

**Dan Zhu** received an M.S. in Physics from Hubei University, in 1986, and a Ph.D. in Physics and Electronics in 2001. After having completed a postdoctoral fellowship in Biomedical Engineering at Huazhong University of Science and Technology (HUST), she obtained the position of Associate Professor in Biomedical Engineering in 2003, and Professor in 2007. Now, she serves as Professor of Wuhan National Opto-Electronics Laboratory, HUST. During recent years, she has been focused on optical clearing of tissue in vivo. She is also the Secretary General of the Biomedical Photonics Committee of the Chinese Optical Society.

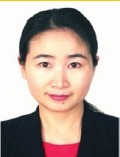

**Kirill V. Larin** received a first M.S. degree in laser physics and mathematics from the Saratov State University, Saratov, Russia, in 1995, a second M.S. degree in cellular physiology and molecular biophysics in 2001, and a Ph.D. degree in biomedical engineering in 2002 from the University of Texas Medical Branch, Galveston. He is an Associate Professor of Biomedical Engineering at the University of Houston, Houston, TX. His research interests include biomedical optics and biophotonics and development and application of various optical methods for noninvasive and nondestructive imaging and diagnostics of tissues and cells.

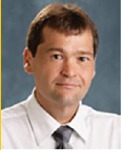

**Qingming Luo** received an M.S. in Techniqical Physics (1966) from Xidian University, and a Ph.D in Physics and Electronics (1993) from Huazhong University of Science and Technology (HUST), China. After having completed his postdoctoral fellowship in Britton Chance Lab, University of Pennsylvania, USA, he set up the biomedical optical research center in HUST in 1997, and has been the head of Key Biomedical Optics Laboratory of Ministry of Education since 2000. Also, since 2007, he has been serving as Executive director of Wuhan National Laboratory of Optoelectronics, vice president of HUST. His research areas include Biomedical Optics, an interdisciplinary research field of Informative Optoelectronics and Biomedicine.

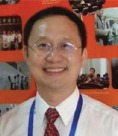

**Valery V. Tuchin** received an M.S. in Radio-Physics and Electronics (1966), a Ph.D. in Optics (1974), and a Dr.Sc. (1982) from the Saratov State University, Saratov, Russia. Currently he is a Professor and holds the Optics and Biophotonics Chair. He is also a Director of the Research-Educational Institute of Optics and Biophotonics at Saratov State University and Head of Laboratory on Laser Diagnostics of Technical and Living Systems, Inst. of Precise Mechanics and Control, RAS. His research interests include biophotonics, biomedical optics and laser medicine, physics of optical and laser measurements. In 2007 he was awarded the SPIE Educator Award.